# Expression of non-phosphorylatable S5A-L-plastin exerts phenotypes distinct from L-plastin deficiency during podosome formation and phagocytosis

**DOI:** 10.3389/fcell.2023.1020091

**Published:** 2023-04-17

**Authors:** Xue Lin, Praveen Krishnamoorthy, Emma C. Walker, Hemant Joshi, Sharon Celeste Morley

**Affiliations:** ^1^ Department of Pediatrics, Division of Infectious Diseases, Washington University School of Medicine, St. Louis, MO, United States; ^2^ Washington University Center for Cellular Imaging, Washington University School of Medicine, St. Louis, MO, United States; ^3^ Department of Pathology and Immunology, Division of Immunobiology, Washington University School of Medicine, St. Louis, MO, United States

**Keywords:** L-plastin, actin cytoskeletal remodeling, macrophage, podosome, phagocytosis

## Abstract

**Introduction:** The actin cytoskeleton remodels to enable diverse processes essential to immunity, such as cell adhesion, migration and phagocytosis. A panoply of actin-binding proteins regulate these rapid rearrangements to induce actin-based shape changes and to generate force. L-plastin (LPL) is a leukocyte-specific, actin-bundling protein that is regulated in part by phosphorylation of the Ser-5 residue. LPL deficiency in macrophages impairs motility, but not phagocytosis; we recently found that expression of LPL in which the S5 residue is converted to a non-phosphorylatable alanine (S5A-LPL) resulted in diminished phagocytosis, but unimpaired motility.

**Methods:** To provide mechanistic insight into these findings, we now compare the formation of podosomes (an adhesive structure) and phagosomes in alveolar macrophages derived from wild-type (WT), LPL-deficient, or S5A-LPL mice. Both podosomes and phagosomes require rapid remodeling of actin, and both are force-transmitting. Actin rearrangement, force generation, and signaling rely upon recruitment of many actin-binding proteins, including the adaptor protein vinculin and the integrin-associated kinase Pyk2. Prior work suggested that vinculin localization to podosomes was independent of LPL, while Pyk2 was displaced by LPL deficiency. We therefore chose to compare vinculin and Pyk2 co-localization with F-actin at sites of adhesion of phagocytosis in AMs derived from WT, S5A-LPL or LPL^−/−^ mice, using Airyscan confocal microscopy.

**Results:** As described previously, podosome stability was significantly disrupted by LPL deficiency. In contrast, LPL was dispensable for phagocytosis and was not recruited to phagosomes. Recruitment of vinculin to sites of phagocytosis was significantly enhanced in cells lacking LPL. Expression of S5A-LPL impeded phagocytosis, with reduced appearance of ingested bacteria-vinculin aggregates.

**Discussion:** Our systematic analysis of the regulation of LPL during podosome vs. phagosome formation illuminates essential remodeling of actin during key immune processes.

## Introduction

Immune cells rely on rapid rearrangements of the actin cytoskeleton to support multiple effector functions, including migration to sites of infection and phagocytosis of pathogens (Jaumouillé and Waterman, 2020). Disruption of actin-mediated adhesion, motility, or phagocytosis can result in immunodeficiency (Sun et al., 2022). While some actin-binding proteins, such as the Wiskott-Aldrich syndrome protein, have been extensively studied (Ngoenkam et al., 2021), the structure and function of others, such as L-plastin (LPL), have only recently come under scrutiny.

LPL is an actin-bundling protein required for motility and activation of multiple immune cells (Todd et al., 2016; Wabnitz et al., 2016; Xu et al., 2016; Zhou et al., 2016; Joshi et al., 2022). The three plastin isoforms expressed in mammals include I-plastin, with tissue restriction to the intestine and kidney; T-plastin, with a broad tissue distribution; and LPL, expressed normally in hematopoietic cells but may also be ectopically expressed in cancer cells of several origins, including breast, prostate, and colon cancer (Delanote et al., 2005; Samstag and Klemke, 2007; Morley, 2012). All three plastins share homologous actin-bundling C-terminal domains. L-plastin is distinguished from I- and T-plastin by its N-terminal, regulatory “headpiece.” The N-terminal domain of LPL contains two serine residues, at positions 5 and 7, that can be phosphorylated downstream of multiple receptors, such as the TNF-α receptor or lymphocyte antigen receptors (Lin et al., 1998; Freeley et al., 2012; Chellaiah et al., 2018; Chellaiah, 2021).

Serine-5 phosphorylation of LPL was previously reported to be associated with motility and effective T cell receptor signaling (Wabnitz et al., 2007; Wabnitz et al., 2010; Wabnitz et al., 2011; Freeley et al., 2012). To fully define the physiological requirement for LPL Ser-5 phosphorylation, we generated a mouse line with a single amino acid substitution at the endogenous LPL locus, converting Ser-5 to the non-phosphorylatable Ala-5 (Anaya et al., 2021). Mice homozygous for this substitution are referred to as “S5A” mice. In our initial characterization, lymphocyte activation and motility appeared grossly intact. In contrast to macrophages harvested from LPL^−/−^ mice, splenic S5A-LPL macrophages exhibited defective phagocytosis but apparently intact podosome formation (Zhou et al., 2016; Anaya et al., 2021). To define potential mechanisms for these observations, we analyzed phagocytosis and podosome formation in primary alveolar macrophages (AMs) obtained from LPL^−/−^, S5A, or matched wild-type (WT) control mice by high-resolution confocal imaging.

Podosomes are conical, actin-rich structures composed of an F-actin core surrounded by integrin-associated proteins and kinases, such as vinculin, talin, paxillin, FAK/Pyk2, and Src family kinases (Gaidano et al., 1990; Bowden et al., 1999; Pfaff and Jurdic, 2001; Buccione et al., 2004; Block et al., 2008). Podosomes support multiple physiological events, such as cell migration and adhesion, in macrophages (Veselý et al., 2006; Carman et al., 2007). The microscopic visualization of macrophage podosomes can vary from “punctae” to the classic “ring-and-core” structure to larger-order podosome clusters, depending on the macrophage lineage, culture media/stimulation, culture duration, and imaging modality used (Hu et al., 2022). A recent study of podosome formation in primary murine peritoneal macrophages, using 3D-STORM imaging, revealed that culturing macrophages for 4 h after isolation resulted in small, punctae-like podosome structures that matured to the classic ring-and-core appearance after 18 h of *in vitro* culture (Hu et al., 2022). Furthermore, Hu et al. demonstrate that podosomes are not simple 2-D structures, but rather 3-dimensional, mesoscale organelles (Hu et al., 2022).

Interestingly, macrophages may also form transient, podosome-like structures during phagocytosis (Herron et al., 2022). During Fcγ-induced phagocytosis, podosome-like structures, consisting of an F-actin core surrounded by rings of talin, paxillin and myosin, formed at the sites of “frustrated” phagocytosis. These “phagocytic podosomes” were visualized using a combination of iPALM, TIRF-SIM, 3D-SIM, and conventional PALM/STORM imaging modalities in a macrophage cell line (RAW 264.7) expressing a variety of fluorescently-labeled proteins (Herron et al., 2022). Phagocytic podosomes appeared as “hourglass” shaped, about 1 μm in diameter and about 0.4 μm in height (Herron et al., 2022).

We have previously shown that LPL supports podosome formation in primary murine alveolar and peritoneal macrophages, with mislocalization of Pyk2 in the absence of LPL (Todd et al., 2016; Zhou et al., 2016; Joshi et al., 2022). However, expression of S5A-LPL did not appear to impair podosome formation to the same degree (Anaya et al., 2021). Somewhat surprisingly, expression of S5A-LPL appeared to impair phagocytosis, while LPL deficiency did not (Anaya et al., 2021). To further investigate these differences, we chose to examine podosome initiation in parallel with phagocytosis in the same primary macrophage lineage, incubated *in vitro* for the same duration. We chose to study AMs because our primary interest is understanding the pulmonary host immune response to *Streptococcus pneumoniae*. We chose a brief duration of *in vitro* incubation (1 hour) because 1) prolonged incubation with bacteria results in significant cell death, and 2) to ensure AMs maintained intrinsic cell identity after isolation from the alveolar microenvironment.

We focused on the co-localization of LPL, vinculin, and the kinase Pyk2 to podosomes (or podosome-like structures) or to phagosomes. Vinculin is an actin-binding protein that supports cell adhesion, and is a common marker for podosomes (van den Dries et al., 2019). Vinculin is also mechanosensing, and recruitment of vinculin to phagosomes promotes complement-mediated phagocytosis (Jaumouillé et al., 2019). Pyk2 is a tyrosine kinase critical to leukocyte adhesion and motility and is also recruited to podosomes and phagosomes (Yu et al., 2013; Maxson et al., 2018). We found in prior work that vinculin recruitment to adhesion sites appeared independent of LPL, while Pyk2 was mislocalized in LPL-deficient cells (Todd et al., 2016; Joshi et al., 2022). We therefore chose to compare the effects of S5A-LPL expression on vinculin and Pyk2 to those of LPL-deficiency in this study.

By contrasting the co-localization of vinculin and Pyk2 with F-actin during phagocytosis or adhesion in WT, S5A-LPL and LPL-deficient AMs, we sought to identify how LPL may be differentially incorporated by F-actin structures during phagosome or podosome formation. We confirm our prior observations that LPL expression is required for podosome stability but is dispensable for phagocytosis of *S. pneumoniae*. We revealed that neither WT nor S5A-LPL are localized to sites of ingested bacteria. Recruitment of vinculin to sites of phagocytosis was increased in AMs lacking LPL, but appeared diminished in AMs expressing S5A-LPL. Finally, Pyk2 co-localization to podosomes required LPL. Pyk2 recruitment to phagosomes was also disrupted with expression of S5A-LPL. Our study highlights the differential and complex requirements for actin-binding proteins during processes essential for cellular immunity, and demonstrates the need for careful and systematic analysis of their spatiotemporal regulation.

## Materials and methods

### Mice

LPL^−/−^, S5A, and matched wild-type (WT) mice (C57BL/6 background) have been previously described (Chen et al., 2003; Anaya et al., 2021). Validation of the ablation of S5-phosphorylation of LPL in cells derived from S5A mice and of LPL-deficiency in LPL^−/−^ mice was previously demonstrated by immunoblot (Anaya et al., 2021).

Mice were matched for age and sex. Littermate WT mice matched to S5A mice are denoted as “WT,” while the wild-type animals of the same breeding lineage as LPL^−/−^ mice are denoted as “B6.” While all mice are back-crossed to the C57BL/6 background, they have been bred as separate lineages in the animal facility and we have found that each CRISPR lineage is best controlled by littermate WT mice. For instance, the percentage of splenic red pulp macrophages in WT mice was 3% while only 1.5% in B6 controls, and phagocytic activity varied among cells isolated from the B6 mice more than those isolated from WT animals (Anaya et al., 2021).

All animal procedures were conducted in accordance with the National Institute of Health (NIH) Guide for the Care and Use of Laboratory Animals, and approved by the Institutional Animal Care and Use Committee (IACUC), Washington University, St. Louis.

### Antibodies

Antibodies used for imaging include: anti-Pyk2 (Santa Cruz Biotechnology, Inc.; cat#: sc-393181), anti-vinculin (Santa Cruz Biotechnology, Inc.; cat#: sc-25336), anti-L-plastin (Santa Cruz Biotechnology, Inc.; cat#: sc-133218), and anti-Tks5 (Proteintech; Cat#: 18976-1-AP) and anti-phalloidin IFluor 647 (Abcam; cat#: ab176759).

Primary antibodies used for Western blot include: anti-Pyk2 (Cell Signaling; cat#: 3292S), anti-vinculin (Santa Cruz Biotechnology, Inc.; cat#: sc-25336), and anti-beta actin (Cell Signaling; cat#: 8457S). Anti-L-plastin was provided by Eric J. Brown, Genentech (Morley et al., 2010; Wang et al., 2010). Specificity of anti-LPL antibodies has been validated by immunofluorescent microscopy using LPL-deficient cells Supplementary Figure S1 (Morley et al., 2010); and by immunoblot (Anaya et al., 2021; Joshi et al., 2022). Specificity of anti-Pyk2, anti-actin and anti-vinculin were confirmed by visualization of single protein band in immunoblots (Supplementary Figure S2).

### Cell isolation

To obtain alveolar macrophages (AMs) bronchoalveolar lavage (BAL) was harvested from naïve mice using DPBS (gibco; Cat#14190-136) containing 1% fetal bovine serum (FBS) (Sigma-Aldrich; Cat#F2442) and 250 μM EDTA. BAL was re-suspended in complete RPMI-1640 (gibco; Cat#11835-030) for further stimulations and experiments. AMs were incubated on coverslips immediately after isolation, and exposure to serum or *S. pneumoniae* was performed on the coverslips. As a control for how AMs would appear after 60 min of culture after isolation, but without specific stimulation, replicate samples were incubated in parallel with AMs undergoing serum stimulation or infection with *S. pneumoniae*.

### Serum stimulation

Synchronous podosome formation was induced by culturing AMs in media with 1% FBS for 30 min (termed “serum starvation”) and then re-cultured in media with 10% FBS for an additional 30 min (serum stimulation).

### Phagocytosis

Freshly isolated AMs were incubated with fluorescently-labeled *S. pneumoniae* (strain D39; serotype 2) for 60 min prior to fixation. WT D39 bacteria was kindly provided by Dr. J. Rosch (Rowe et al., 2019). Fluorescently-labeled D39 (either GFP or RFP) were generated as described (Kjos et al., 2015). Plasmids containing a chloramphenicol resistance gene (*cat*) and either superfolder green fluorescent protein (GFP; *sfgfp* (*Bs*)) or far-red fluorescent protein (RFP; mKate2) fused to the abundant histone-like protein HlpA (*hlpA*), were graciously provided by Jan-Willem Veening. D39 was transformed by growing in Todd Hewitt Broth + 0.2% yeast (THB + Y) extract to an optical density at 600 nm (OD_600_) of 0.1, incubating at 37°C for 12 min with competence-stimulating peptide 1 (CSP-1; 100 ng/mL), then incubating with constructs expressing GFP or RFP (described above) for an additional 20 min at 30°C. Bacteria were diluted 1:10 in THB + Y and incubated for 90 min at 37°C. Transformants were selected by plating on blood agar plates with chloramphenicol (2.5 μg/mL). Fluorescence of bacteria was confirmed through microscopic examination and flow cytometry.

### Confocal microscopy

Cells were fixed with 4% paraformaldehyde, permeabilized with 0.2% Triton™ X-100, then stained with Phalloidin-AlexaFluor-647 (working solution prepared based on the manufacturer’s instructions; for F-actin) and/or conjugated (AF488, AF594, or AF647) anti-Pyk2 antibody (1:100), anti-vinculin (1:100), or anti-L-plastin (1:100); nuclei were visualized with DAPI. For Tks5 staining, after fixation and permeabilization, the cells were stained with primary anti Tks5 (1:100), then followed by Phalloidin-AlexaFluor-647, conjugated (AF488 or AF594) anti-Pyk2 antibody (1:100), anti-vinculin (1:100), or anti-L-plastin (1:100), and the secondary antibody used for anti-Tks5 visualization (Alexa Fluor 405, goat anti-Rabbit IgG-AF405; Invitrogen; Cat#: A31556; 1:100). The secondary antibody used for anti-Tks5 visualization was Alexa Fluor 405, goat anti-Rabbit IgG-AF405 (Invitrogen; Cat#: A31556; 1:100). Nuclei were visualized with DAPI. Images were acquired using a Zeiss LSM 880 II with Airyscan FAST confocal microscope with a ×60 oil objective. Each ‘slice’ for z-stacks had a thickness of approximately 0.15—0.18 μm. For each condition, all cells apparent in six randomly selected fields were acquired and analyzed. Z-stacks in figures are visualized primarily as 2D XY projections in images, generated using the Imaris Image Processing function 2D Projection → XY plane. Imaris was also used to reconstruct 3D images of cells from acquired z-stacks, provided in some figures and as Supplementary Videos.

### Image analysis

**FIGURE 1:**
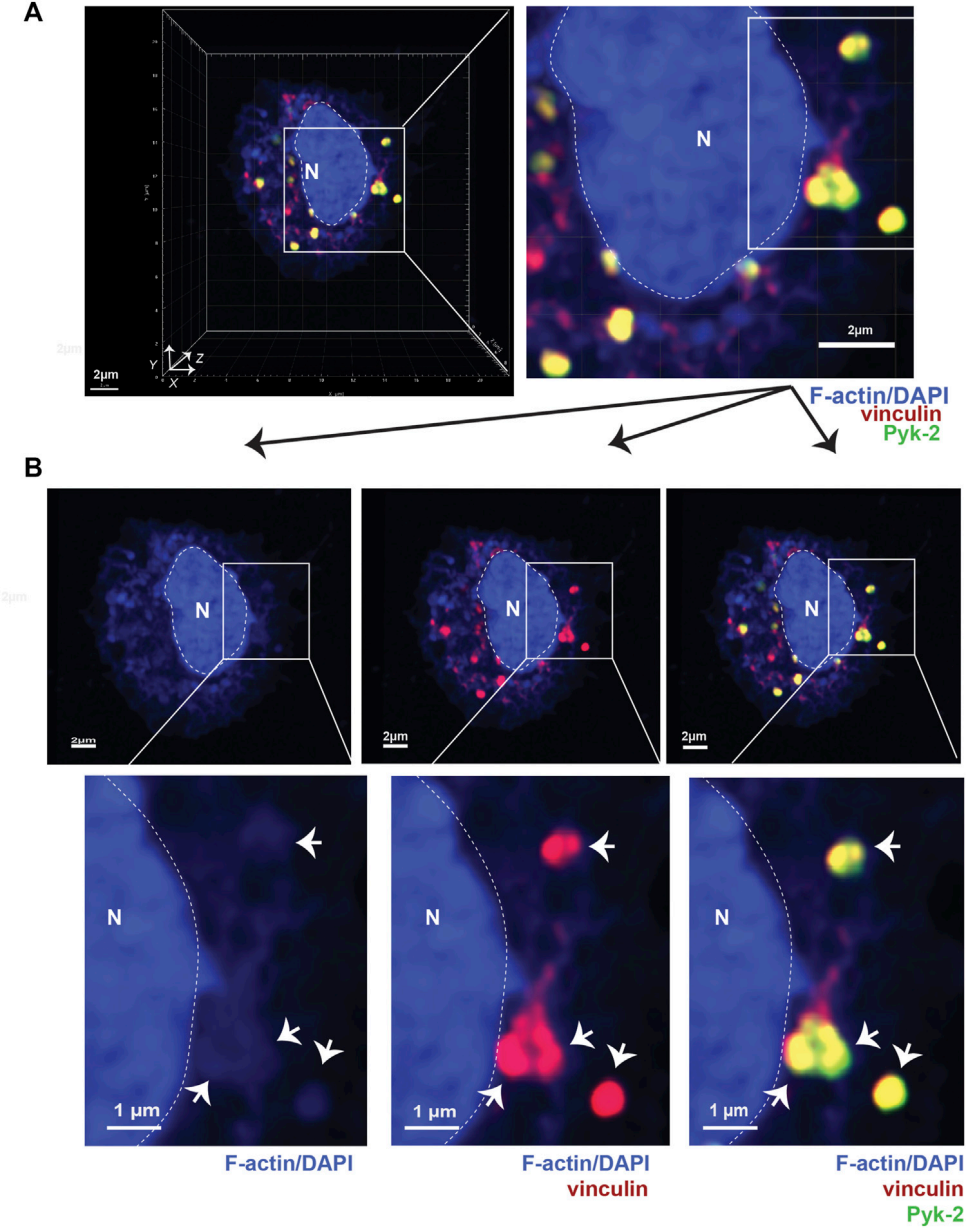
Podosomes are three-dimensional structures that include vinculin and Pyk2. **(A)** Airyscan confocal microscopy was used to acquire z-stacks of fixed AMs to visualize cellular location of F-actin (blue), vinculin (red), and Pyk2 (green). DAPI was used to image nucleus. (“N” and dotted line). Imaris software was used to reconstruct 3D image of cell, shown in orthogonal view. Grid shows 2 μm increments and is optimal for size assessment. **(B)** Representative 2D X-Y projection of AM in panel A, showing overlay of Pyk2 and vinculin with F-actin in podosomes (arrows). DAPI was used to image nucleus. (“N” and dotted line). Scale bars derived from grid in “A.”

For quantitatively analyzing protein expression from confocal images, we used Imaris Surface program to precisely visualize and measure the volume of individual and co-localized proteins that were 3D-reconstructed through Z-stack images. Because podosomes and phagosomes are 3D structures [Figure 1; Supplementary Movie S1 (Herron et al., 2022; Hu et al., 2022; Weber et al., 2022)] we chose to reconstruct 3D projections of AMs using the new Imaris Surface program. Recent work by Hu et al. suggests that nascent podosomes have a minimum radius of approximate 250 nm (0.25 μm) and a height of 350 nm (0.35 μm), which provides an approximate volume calculation of 0.07 μm^3^. Imaris Surface includes a new tool to calculate the volume of overlap of two signals. By applying this tool to each cell, we can objectively quantify protein aggregate volumes, which is a new way of analyzing F-actin based structures. Applying the quantification to each cell avoids subjective selection of individual podosomes. Pearson’s coefficient was calculated using ImageJ/Fiji software. Scale bars were exported on orthogonal z-stacks from Imaris, with “grid lines” spaced at 2 μm increments. Subsequent panels show scale bars calculated from 2 μm grid lines resized proportional to cells shown.

### Immunoblots

Immunoblots were performed as described (Todd et al., 2011). Membranes were probed with the indicated primary antibodies and beta-actin (D6A8) as loading control. Goat anti-rabbit-AlexaFluor680 (Invitrogen) and goat anti-mouse-IRDye800 (Rockland Immunochemicals, Gilbertsville, PA) were used to detect membrane-bound antibodies. Signal was visualized using the LiCOR Odyssey imaging system and software (LiCOR, Lincoln, NE).

### Statistics and display of data

Data tables and graphs were generated using GraphPad Prism (v 9.0). Scale bars for microscopy images were generated in Imaris software and exported as *.png files. Figures were assembled using Adobe Illustrator (v 2021, Adobe, San Jose, CA), with integrity of scaling and resolution maintained. For Tks5 staining only, the Tks5 staining was overlaid as a separate layer with 50% transparency on the combined F-actin + Pyk2+vinculin or F-actin + LPL + Pyk2 image (Adobe PhotoShop, 2021) to clearly display co-localization of signals. Statistical significance was determined using Mann-Whitney (non-parametric) tests, with a value of *p* < 0.05 considered significant.

## Results

1. Following serum stimulation, podosome stability requires LPL expression

To better detail how LPL and S5A-LPL support podosome formation, we induced synchronous podosome initiation in freshly isolated AMs from WT, S5A, B6 and LPL−/− mice by incubating cells in medium containing 1% FBS for 30 min, followed by incubation in medium with 10% FBS for an additional 30 min. We then imaged full-thickness z-stacks of AMs stained to illuminate F-actin, vinculin, and Pyk2 using Airyscan confocal microscopy. Co-localization of Pyk2, vinculin and F-actin in AMs was then reconstructed in three dimensions from acquired Z-stacks (Figure 1A). Nascent podosomes were defined as discrete co-localizations (punctae) of F-actin, vinculin and Pyk2, many of which extended from the basal to the apical surface of the cell (Figure 1A, Supp. Movie 1). For illustration, 2D X-Y projections of representative cells are provided to show co-localization of the indicated proteins (Figure 1B). Classification of these structures as nascent podosomes in WT AMs was confirmed by illumination with anti-Tks5 staining (Supplementary Figure S4). Tks5 is a scaffold protein used as a marker of invadopodia and podosomes (Di Martino et al., 2014). The size (0.5—1 μm in diameter) and appearance (“punctae”) of these nascent podosomes is consistent with prior reports of primary macrophages incubated for relatively short durations (e.g., 4 h) *in vitro*. For quantitative analysis in subsequent experiments, the total volumes of the indicated aggregates are provided, which better accounts for the 3-D structure of podosomes.

**FIGURE 2:**
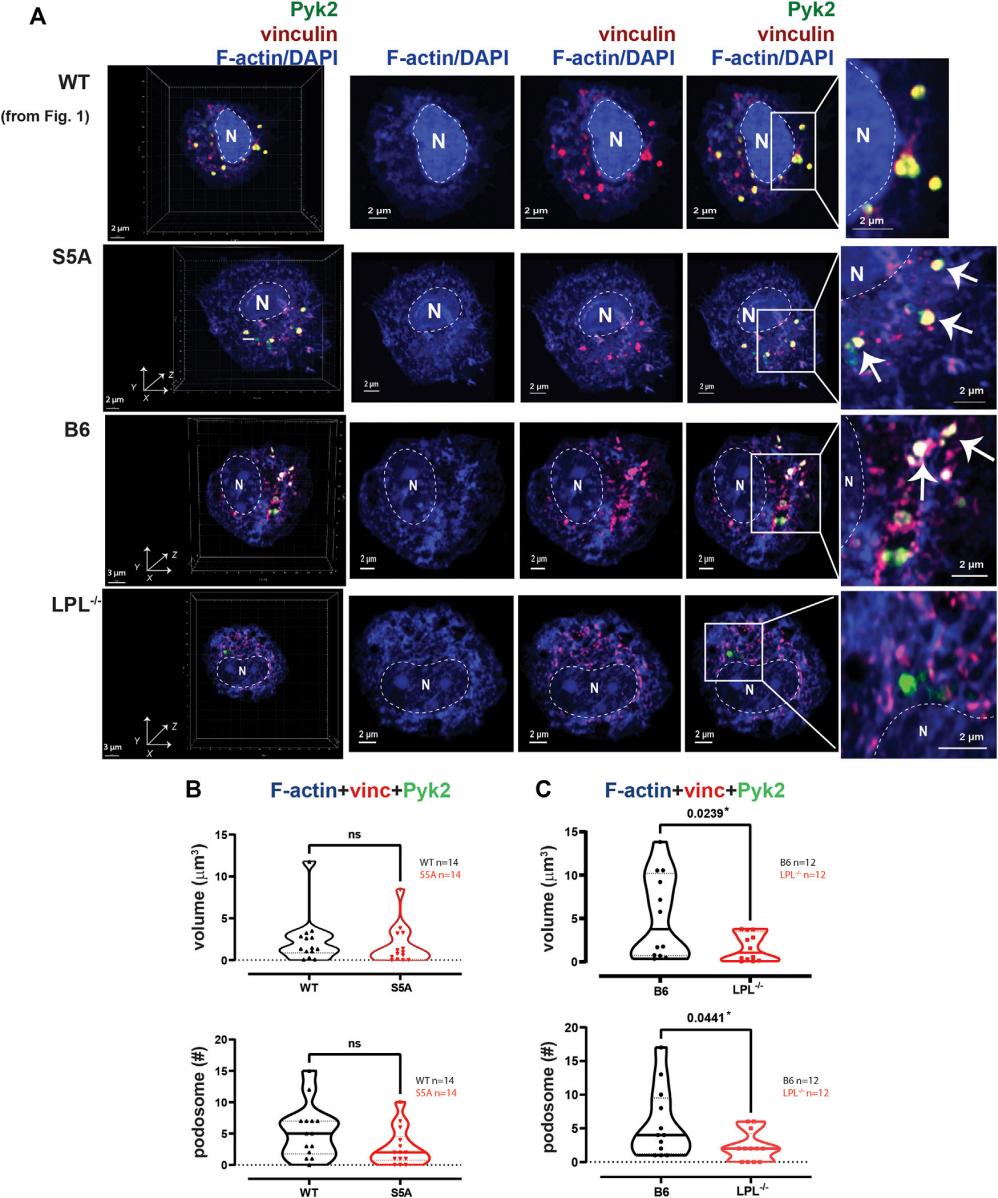
Podosome formation after serum stimulation requires LPL. **(A)** Co-localization of Pyk2 (green), vinculin (red) and F-actin (blue) in serum-stimulated AMs from matched WT and S5A or B6 and LPL^−/−^ mice. Far left panels show 3D reconstruction of Z-stack confocal micrographs. The “WT” cell (top row) is the same cell shown in Figure 1. Overlays of vinculin with F-actin and of Pyk2 with vinculin + F-actin are shown to indicate how “volumes” are derived in subsequent analyses. Podosomes are aggregates in which all three proteins are co-localized (arrows). Images shown are 2D XY projections of the z-stacks. DAPI was used to image nucleus (“N” and dotted line). Grids show 2 μm increments and are optimal for size assessment; scale bars in subsequent panels are derived from grids. **(B)** Volumes of aggregates of co-localized F-actin + vinculin + Pyk2, and the number of aggregates ≥ 0.07 μm^3^ in AMs from matched WT and S5A mice. **(C)** Volumes of aggregates of co-localized F-actin + vinculin + Pyk2, and the number of aggregates ≥ 0.07 μm^3^ in AMs from matched B6 and LPL^−/−^ mice. **(B, C)** Each symbol represents the volume calculated from one AM; n = number of cells analyzed. Images acquired from two independent experiments. *p*-values determined by Mann-Whitney; ns = non-significant.

We have shown in prior work that LPL is not required for initiation of podosome formation (within 5 min of contact with surface), but is essential to maintain podosome stability, with substantial loss of podosomes after 30 m of incubation (Zhou et al., 2016). To interrogate if Ser-5 phosphorylation of LPL was also required for podosome stability, we analyzed AMs from S5A, LPL^−/−^ and matched WT or B6 control mice following serum starvation and re-exposure to serum (“serum stimulation”) (Figure 2; Supplementary Movies S1–S4; Supplementary Figure S4; the WT AM image in Figure 2A is the same cell used for exemplary purposes in Figure 1). Exposure to serum after brief starvation induces the rapid and synchronous formation of new podosomes (Veselý et al., 2006).

To quantify nascent podosomes, we visualized F-actin, vinculin, and Pyk2 in AMs expressing LPL, S5A-LPL or deficient for LPL (Figure 2A). Imaris Surface software was used to visualize (Figure 2A, arrows) and measure (Figures 2B, C) the volume of aggregates of co-localized proteins. Based upon prior published work, nascent podosomes were defined as aggregates of F-actin, vinculin, and Pyk2 of at least 0.07 μm^3^ (Linder, 2007; Wiesner et al., 2014; Hu et al., 2022). We confirmed identity of these structures as nascent podosomes through co-localization with Tks5 in AMs from WT and S5A mice (Supplementary Figure S4); AMs from LPL^−/−^ mice did not form a sufficient number of nascent podosomes to visualize Tks5 co-localization. S5A-LPL AMs exhibited similar volumes and numbers of nascent podosomes as matched WT AMs after serum stimulation (Figure 2B). We note that while it may initially appear that there might be a reduction in nascent podosome numbers in AMs derived from S5A mice, this difference was not significantly different and was not seen in subsequent analyses (*e.g.*, definition of nascent podosomes using co-localization of F-actin, vinculin and LPL). In contrast, LPL deficiency significantly reduced both podosome volumes and numbers (Figure 2C). We therefore confirm our prior findings in a rigorous, side-by-side comparison of LPL-deficient and S5A-LPL AMs (Zhou et al., 2016).

**FIGURE 3:**
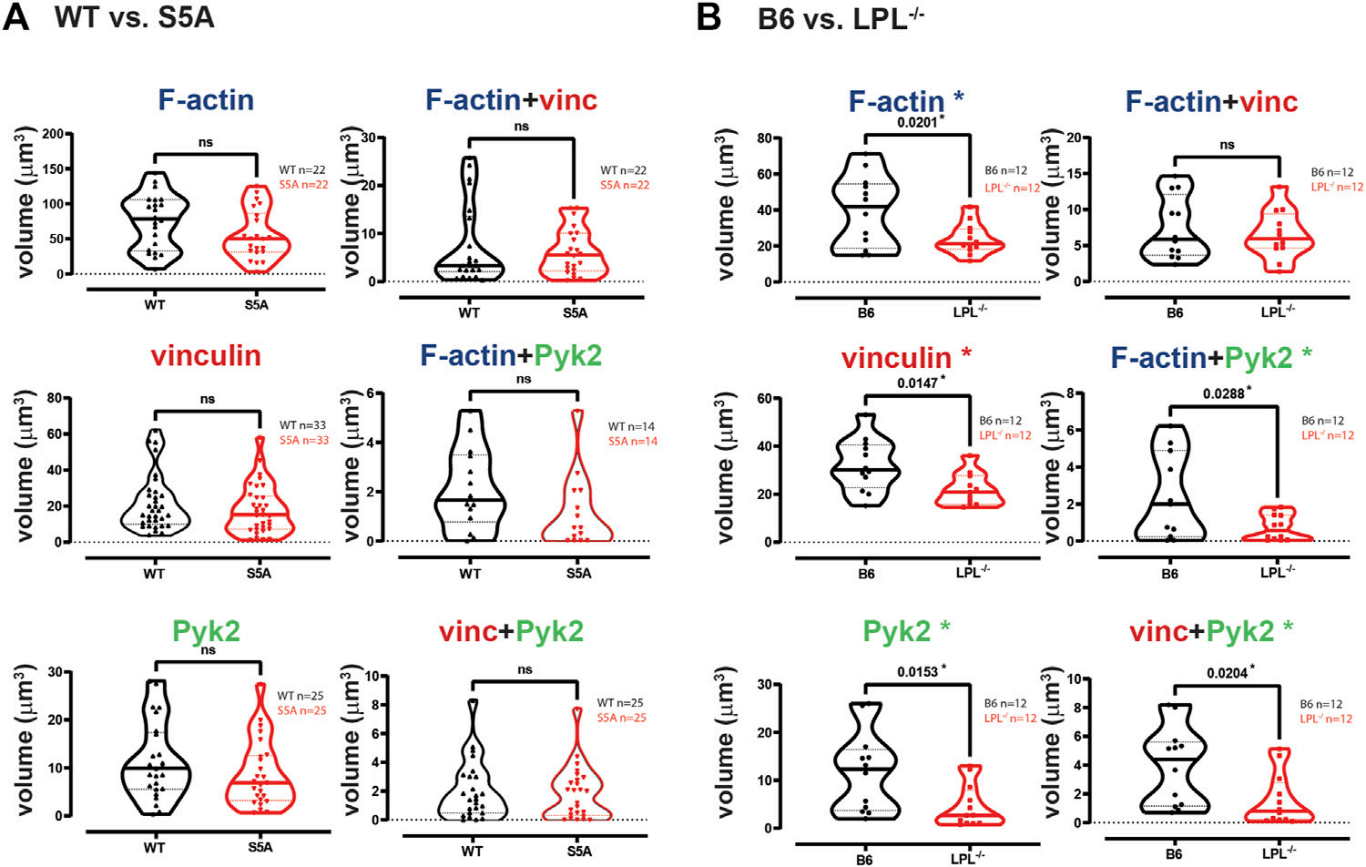
Quantification of volumes of protein aggregates reveals loss of Pyk2 from podosomes in AMs lacking LPL. Imaris software was used to calculate the total volume (μm^3^) of the indicated protein 3D aggregates (images in Figure 2) contributing to podosome formation after serum stimulation of AMs derived from **(A)** matched WT and S5A mice or **(B)** matched B6 and LPL^−/−^ mice. Each symbol represents the volume calculated from one fixed cell; n = number of cells analyzed. Images acquired from two independent experiments. *p*-values determined by Mann-Whitney; ns = non-significant, * = *p* < 0.05.

To systematically analyze co-localization of vinculin and Pyk2 with F-actin structures in three-dimensions, we measured the individual volumes of F-actin, Pyk2 and vinculin, and the co-localized paired volumes of F-actin + vinculin, F-actin + Pyk2, and vinculin + Pyk2. Consistent with similar volumes and numbers of podosomes after serum stimulation, the volumes of individual or paired protein aggregates were similar in WT and S5A-LPL AMs (Figure 3A). In contrast, individual aggregates of F-actin, Pyk2 and vinculin were all reduced in AMs deficient for LPL, as were aggregates of co-localized F-actin + Pyk2 and vinculin + Pyk2. LPL deficiency did not alter the volume of vinculin co-localized with F-actin (Figure 3B). Notably, immunoblot analysis revealed equivalent amounts of vinculin, Pyk2 and actin in AMs from WT, S5A and LPL^−/−^ mice; thus, reduced volumes of aggregates were not due to reduced protein expression (Supplementary Figure S3). Analysis of nascent podosomes in AMs lacking LPL is therefore consistent with prior observations that co-localization of vinculin to F-actin in podosomes may occur independently of LPL, but co-localization of Pyk2 is disrupted (Zhou et al., 2016; Joshi et al., 2022).

**FIGURE 4:**
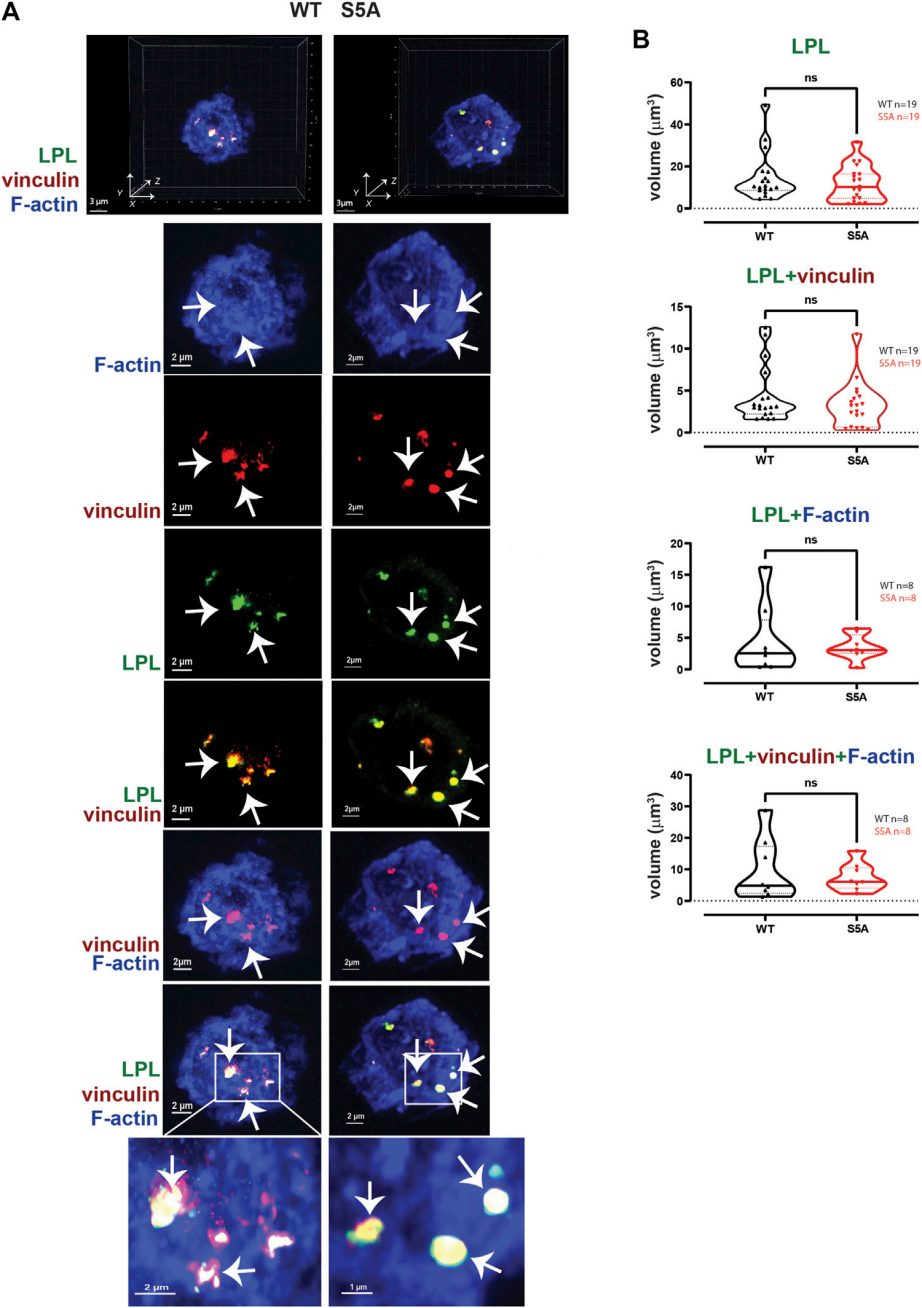
WT-LPL or S5A-LPL co-localize equivalently with vinculin and/or F-actin in AMs after serum stimulation. **(A)** Co-localization of LPL (green), vinculin (red) and F-actin (blue) in serum stimulated AMs from matched WT and S5A mice. Images in top panels show 3D reconstruction of Z-stack confocal micrographs. Other panels are 2D XY projections showing individual proteins and overlays of LPL + vinculin (yellow), vinculin + F-actin (purple), and of LPL + vinculin + F-actin (white). Representative podosomes are indicated by white arrows and magnified in insets. Grids show 2 μm increments and are optimal for size assessment; scale bars in subsequent panels are derived from grids. **(B)** Total volume of aggregates of LPL, LPL + vinculin, LPL + F-actin, and of LPL + vinculin + F-actin. Each symbol represents the volumes calculated from one AM; n = number of cells analyzed. Images acquired from two independent experiments. *p*-values determined by Mann-Whitney; ns = non-significant.

LPL is recruited to nascent podosomes in macrophages, localizing to the core in mature podosomes (De Clercq et al., 2013; Zhou et al., 2016). To determine if S5A-LPL was likewise incorporated into nascent podosomes, we next analyzed the co-localization of LPL with F-actin, vinculin, and/or Pyk2 in AMs derived from WT or S5A mice (Figure 4). We found no significant differences in volumes of LPL, LPL co-localized with vinculin, F-actin, or Pyk2, or of the colocalization of LPL, vinculin and F-actin or of LPL, Pyk2 and F-actin, in AMs expressing S5A-LPL compared to those expressing WT-LPL (Figure 4). We have not yet been able to validate an antibody specific for S5-phosphorylated LPL for immunofluoresence, and therefore cannot assess if S5-phosphorylated LPL is localized to nascent podosomes. However, our data suggest that S5 phosphorylation is not required for inclusion of LPL to nascent podosomes.

**FIGURE 5:**
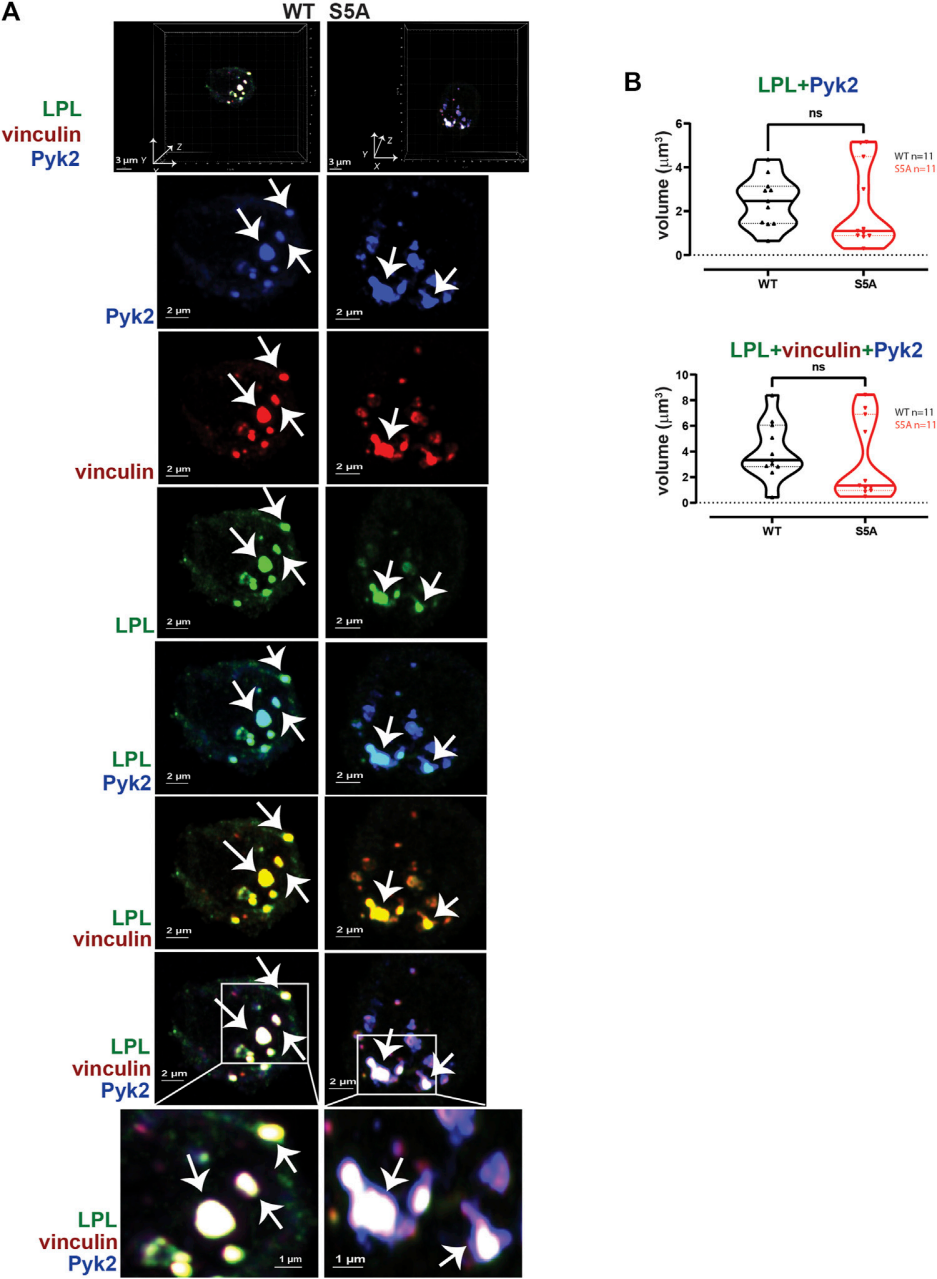
WT-LPL and S5A-LPL co-localize equivalently with Pyk2 and vinculin in AMs after serum stimulation. **(A)** Co-localization of LPL (green), vinculin (red) and Pyk2 (blue) in serum-stimulated AMs from matched WT and S5A mice. Top panel shows 3D reconstruction of Z-stack confocal micrographs. Individual protein staining, overlays of Pyk2+LPL (aqua), LPL + vinculin (yellow), and of LPL + vinculin + Pyk2 (white) are 2D XY projections. Representative podosomes indicated by white arrows. Grids show 2 μm increments and are optimal for size assessment; scale bars in subsequent panels are derived from grids. **(B)** Total volumes of LPL + Pyk2 or LPL + Pyk2+vinculin aggregates shown in **(A)**. Each symbol represents the volume calculated from one fixed cell; n = number of cells analyzed. Images acquired from two independent experiments. *p*-values determined by Mann-Whitney; ns = non-significant.

To test if expression of S5A-LPL altered formation of LPL aggregates with Pyk2 or with vinculin and Pyk2, we measured the volumes of LPL + Pyk2 and LPL + vinculin + Pyk2 aggregates after serum stimulation of AMs from WT or S5A mice. Imaging revealed aggregates of co-localized LPL, vinculin and Pyk2 of similar volumes in AMs expressing WT-LPL and S5A-LPL, suggesting that S5 phosphorylation of LPL is not required for localization of Pyk2 during initiation of nascent podosomes. (Figure 5).

2. Reduced numbers of podosomes in AMs expressing S5A-LPL when cultured after isolation without stimulation

AMs are primary murine macrophages that are exquisitely sensitive to their extracellular environment. Isolation from their *in vivo* environment and maintenance in tissue culture plates induces changes in adhesion, metabolism and proliferation (Subramanian et al., 2022). The rate and degree of these changes is undefined. Because AMs can undergo significant phenotypic changes following isolation and *in vitro* culturing, we analyzed the morphology of AMs derived from WT, S5A, and LPL^−/−^ mice after culturing for 60 min, without specific stimulation. The duration of 60 min was selected so that AM morphology could be compared to those that underwent serum stimulation (30 min serum starvation + 30 min serum re-exposure) immediately after isolation.

**FIGURE 6:**
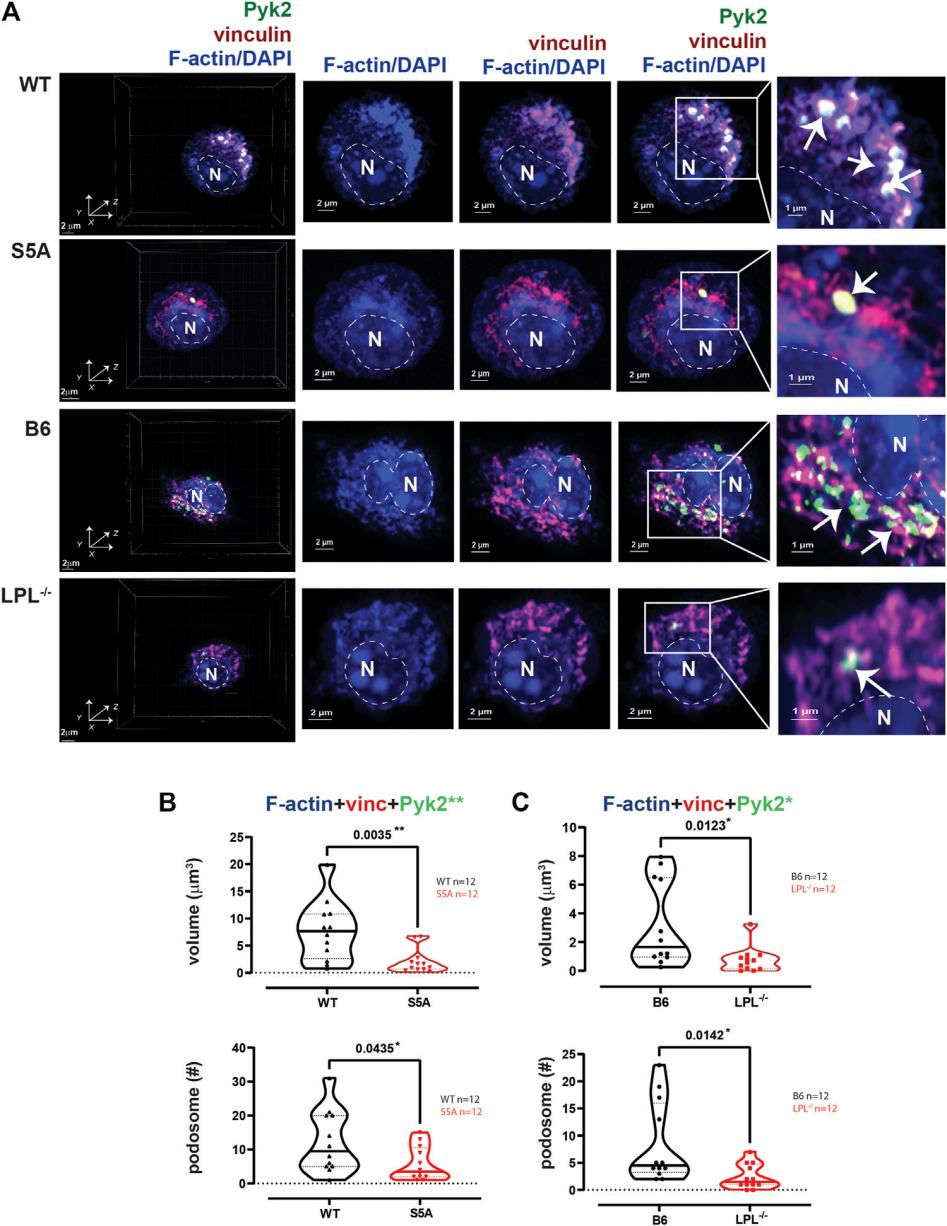
Podosome formation during incubation on coverslips, without additional stimulation, requires S5-phosphorylation of LPL. **(A)** Co-localization of Pyk2 (green), vinculin (red) and F-actin (blue) in AMs derived from matched WT and S5A or B6 and LPL^−/−^ mice after 60 min incubation on coverslips without additional stimulation. 60 min was selected as the duration to correspond the 60 min post-isolation duration of incubation of AMs analyzed after serum stimulation and with Spn infection. Far left panels show 3D reconstruction of Z-stack confocal micrographs. Overlays of vinculin with F-actin and of Pyk2 with vinculin + F-actin are shown to indicate how “volumes” are derived in subsequent analyses. Podosomes are aggregates in which all three proteins are co-localized (arrows), magnified in insets. DAPI was used to image nucleus. (“N” and dotted line). Grids show 2 μm increments and are optimal for size assessment; scale bars in subsequent panels are derived from grids. **(B)** Volumes of aggregates of co-localized F-actin + vinculin + Pyk2, and the number of aggregates ≥ 0.07 μm^3^ in AMs from matched WT and S5A mice. **(C)** Volumes of aggregates of co-localized F-actin + vinculin + Pyk2, and the number of aggregates ≥ 0.07 μm^3^ in AMs from matched B6 and LPL^−/−^ mice. **(B, C)** Each symbol represents the volume calculated from one AM; n = number of cells analyzed. Images acquired from two independent experiments. *p*-values determined by Mann-Whitney; *p < 0.05, **p < 0.01.

AMs from WT and B6 mice formed nascent podosomes when cultured without specific stimulation, with similar volumes and numbers as when stimulated with serum (Figures 2, 6). While the specific stimulus is undefined, adhesion to coverslips is likely sufficient to induce podosomes (Subramanian et al., 2022). In contrast to results obtained following serum stimulation, podosome numbers and volumes were significantly reduced in AMs expressing S5A-LPL when cultured for 60 min after isolation without specific stimulation (Figures 6A, B). Furthermore, co-localization of Tks5 with nascent podosomes appeared to be reduced, consistent with disruption of nascent podosomes (Supplementary Figure S5). AMs without LPL again exhibited reduced podosome volumes and numbers (Figure 6C). Thus, S5-phosphorylation of LPL is required for podosomes under some, but not all, culture conditions.

**FIGURE 7:**
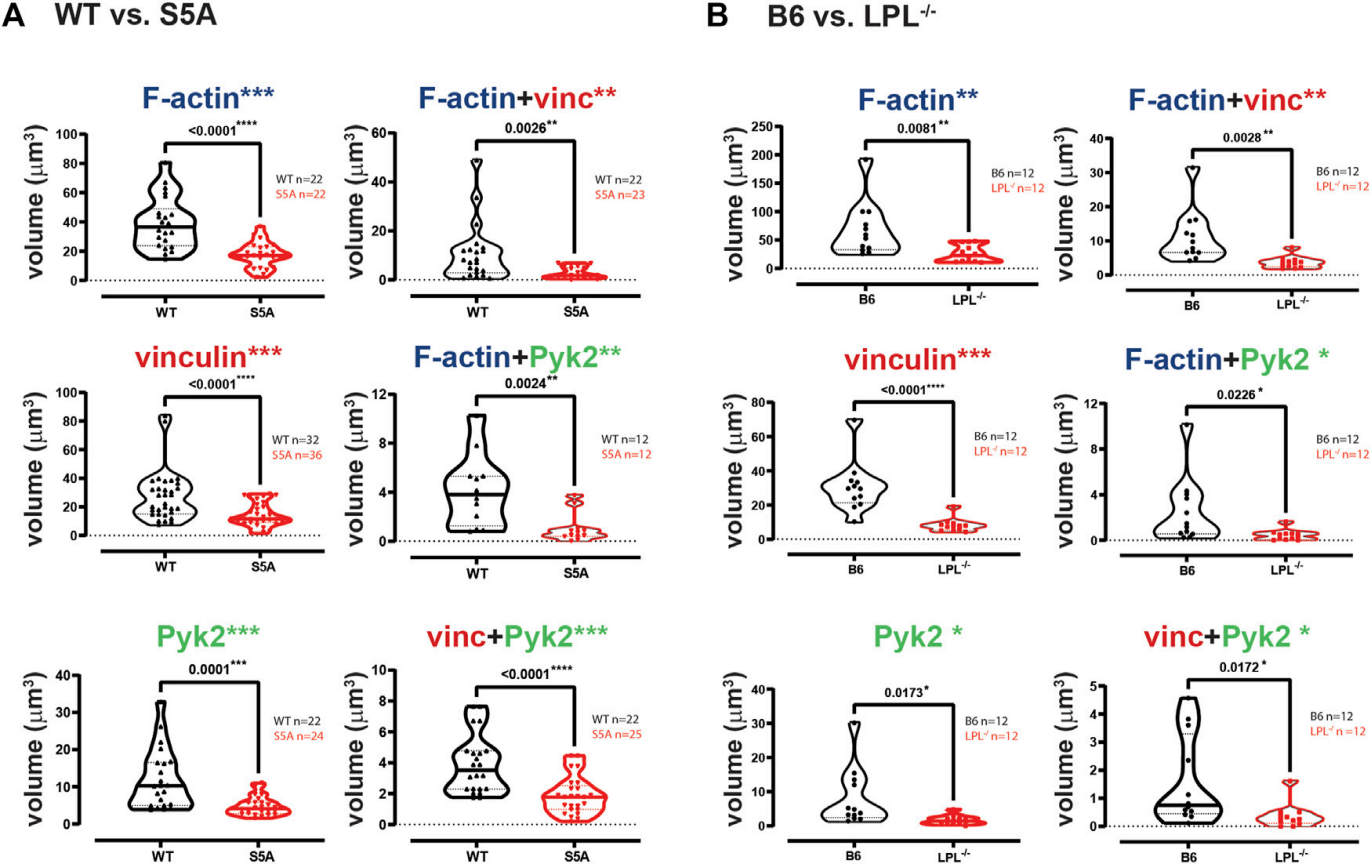
Quantification of volumes of protein aggregates reveals loss of vinculin and Pyk2 from podosomes in AMs expressing S5A-LPL or AMs lacking LPL. Imaris software was used to calculate the total volume (μm^3^) of the indicated protein 3D aggregates contributing to podosome formation after 60 min incubation of AMs derived from **(A)** matched WT and S5A mice or **(B)** matched B6 and LPL^−/−^ mice (Images in Figure 6). Each symbol represents the volume calculated from one fixed cell; n = number of cells analyzed. Images acquired from two independent experiments. *p*-values determined by Mann-Whitney; *p < 0.05, **p < 0.01, ***p < 0.0001.

**FIGURE 8:**
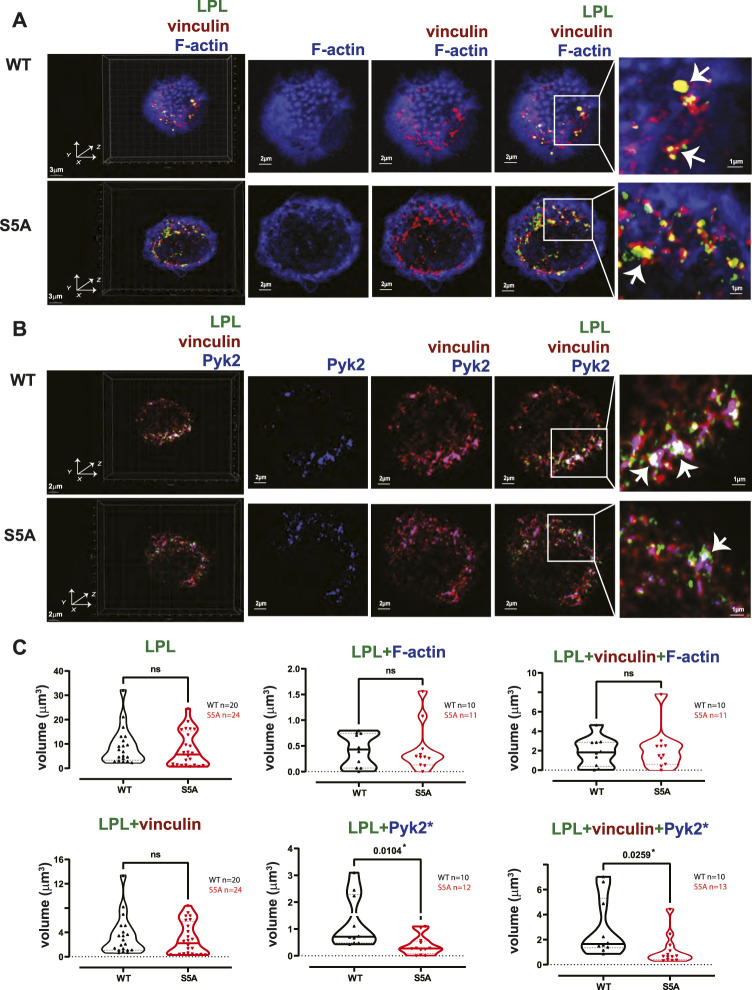
Co-localization of S5A-LPL with Pyk2, but not vinculin, diminished in AMs incubated for 60 min after isolation. **(A, B)** Co-localization of **(A)** LPL (green), vinculin (red) and F-actin (blue) or of **(B)** LPL (green), vinculin (red) and Pyk2 (blue) in AMs from matched WT and S5A mice, imaged after 60 min incubation on glass coverslips without additional stimulation. Images in left panels show 3D reconstruction of Z-stack confocal micrographs. Other panels are 2D XY projections showing F-actin only; overlay of **(A)** vinculin + F-actin (purple), or **(B)** vinculin + Pyk2; or **(A)** overlay of LPL + vinculin + F-actin (white) or **(B)** LPL + vinculin + Pyk2 (white). Representative podosomes are indicated by white arrows and magnified in insets. Grids show 2 μm increments and are optimal for size assessment; scale bars in subsequent panels are derived from grids. **(C)** Total volume of aggregates of LPL, LPL + vinculin, LPL + F-actin, LPL + Pyk2, LPL + vinculin + F-actin, and of LPL + vinculin + Pyk2. Each symbol represents the volumes calculated from one AM; n = number of cells analyzed. Images acquired from two independent experiments. *p*-values determined by Mann-Whitney; ns = non-significant, *p < 0.05.

The individual volumes of F-actin, vinculin, and Pyk2 were significantly decreased in AMs from S5A and LPL^−/−^ mice compared to matched WT or B6 mice (Figure 7). The volumes of co-localized proteins, analyzed in pairs (vinculin + F-actin, F-actin + Pyk2, and vinculin + Pyk2), were also significantly reduced in AMs from S5A and LPL^−/−^ mice (Figure 7). To further explore how S5A-LPL may impair or disrupt podosomes when AMs are cultured without stimulation for 60 m, we quantified LPL aggregates, or aggregates of LPL with vinculin, with F-actin, with Pyk2, or with combinations of vinculin + F-actin and vinculin + Pyk2. Aggregates of LPL alone, LPL + F-actin, LPL + vinculin, and of LPL + vinculin + F-actin were of similar volumes in AMs expressing WT-LPL or S5A-LPL. However, volumes of LPL + Pyk2 and of LPL + vinculin + Pyk2 were significantly reduced when S5A-LPL was expressed (Figure 8). Decreased volumes of aggregates including Pyk2 are consistent with our prior results that LPL is mechanistically involved in either recruiting or retaining Pyk2 at sites of adhesion. Significantly decreased podosome volumes and numbers in AMs from S5A and LPL^−/−^ mice indicate that both expression and Ser-5 phosphorylation of LPL contribute either to podosome initiation or stability when cultured on coverslips in the absence of specific stimulation.

3. Reduced co-localization of vinculin with sites of pneumococcal phagocytosis in macrophages from S5A, but not LPL^−/−^, mice

**FIGURE 9:**
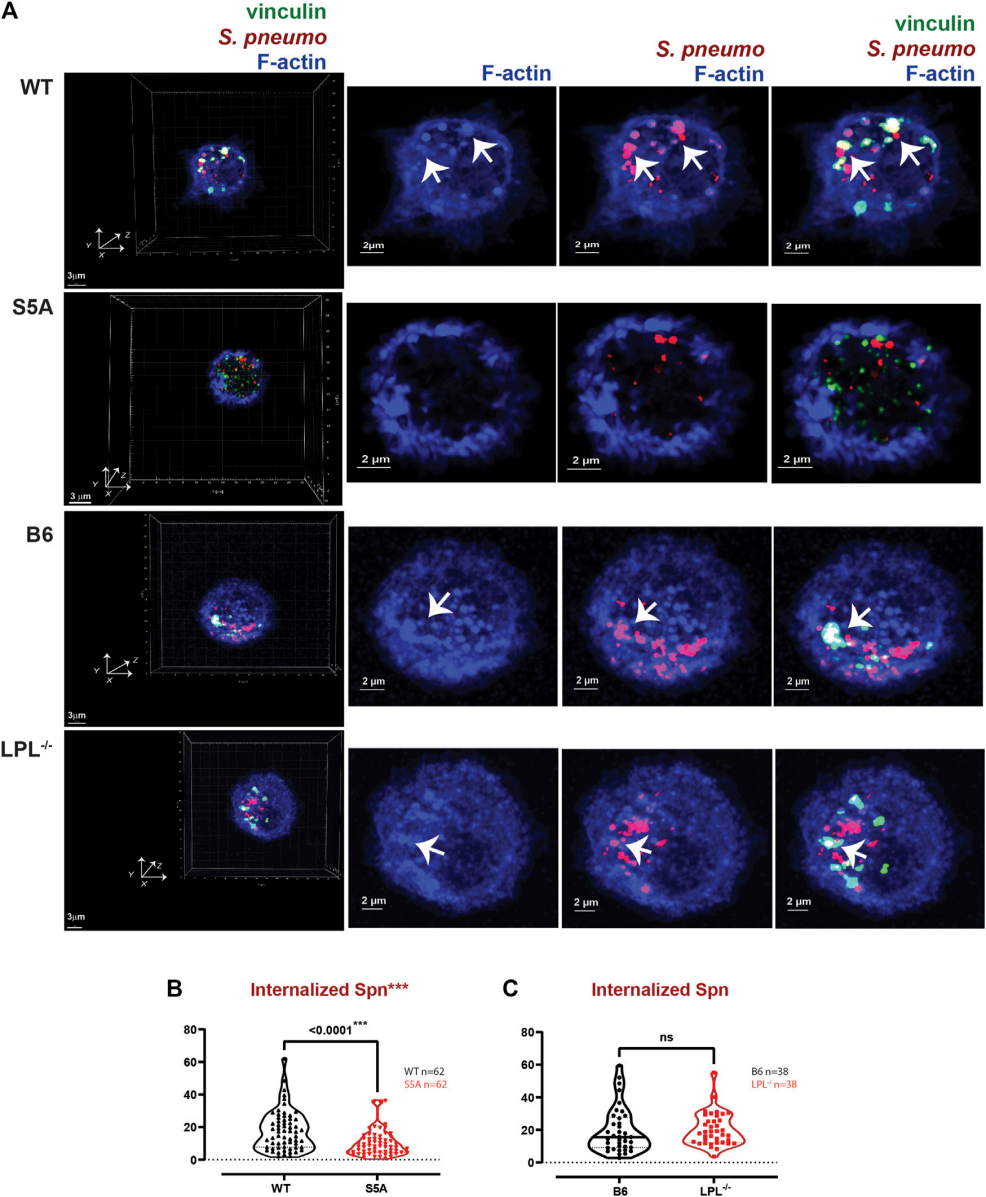
Reduced internalization of Spn by AMs derived from S5A mice. **(A)** Co-localization of vinculin (green), *S. pneumoniae* (red) and F-actin (blue) in AMs incubated with Spn-RFP for 60 min from matched WT and S5A or matched B6 and LPL^−/−^ mice. Images in left panels show 3D reconstruction of Z-stack confocal micrographs. Other panels are 2D XY projections showing F-actin only, Spn overlaid with F-actin, or overlay of vinculin + Spn + F-actin. Phagosomes incorporate F-actin, Spn and vinculin (white; representative phagosomes indicated by white arrows). Grids show 2 μm increments and are optimal for size assessment; scale bars in subsequent panels are derived from grids. **(B, C)** Total volumes of internalized aggregates of Spn, quantifying pneumococcal phagocytosis. Each symbol represents the volume calculated from one fixed cell; n = number of cells analyzed. Images acquired from two independent experiments. *p*-values determined by Mann-Whitney; ns = non-significant; ***p < 0.0001.

In prior work, we found that S5A mice exhibited defective clearance of pneumococcal bacteremia (Anaya et al., 2021), correlating with reduced phagocytosis and a reduced population of splenic marginal zone macrophages. Specifically, both macrophages and neutrophils in S5A mice exhibited phagocytic defects that were not found in LPL^−/−^ mice (Anaya et al., 2021). Given the possible formation of “phagocytic podosomes” in macrophages during phagocytosis, further analysis of the molecular mechanisms underlying the phagocytic defect in cells expressing S5A-LPL is warranted. We therefore co-cultured AMs derived from WT, S5A, B6 and LPL^−/−^ mice with fluorescently-labeled *S. pneumoniae* (Spn-RFP) *in vitro* for 60 min (Figure 9). We found the volume of ingested Spn-RFP in AMs of S5A mice was significantly reduced compared to WT (Figures 9A, B), corroborating our previously observed defect in phagocytosis in S5A mice (Anaya et al., 2021). Consistent with prior data that expression of WT-LPL is dispensable for phagocytosis (Chen et al., 2003; Anaya et al., 2021), we found equivalent volumes of ingested Spn-RFP in AMs derived from B6 or LPL^−/−^ mice (Figures 9A, C). This phenotypic defect when S5A-LPL is expressed, but not when LPL is deficient, suggests that S5A-LPL may act as a “dominant negative.”

**FIGURE 10:**
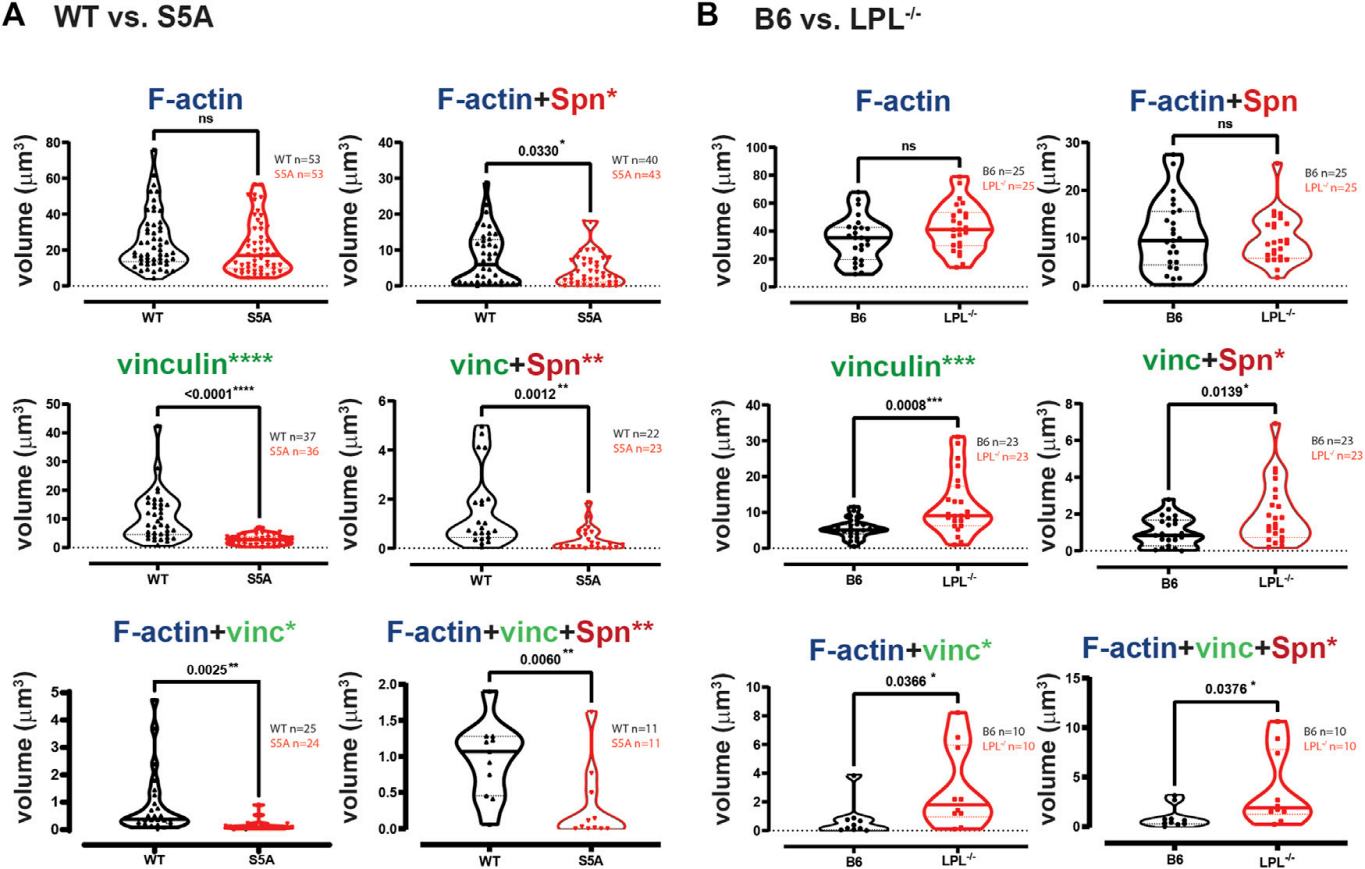
Quantification of volumes of protein aggregates reveals loss of vinculin from phagosomes in AMs expressing S5A-LPL. Imaris software was used to calculate the total volume (μm^3^) of the indicated protein 3D aggregates (images in Figure 9) contributing to pneumococcal phagocytosis by AMs derived from **(A)** matched WT and S5A mice or **(B)** matched B6 and LPL^−/−^ mice. Each symbol represents the volume calculated from one fixed cell; n = number of cells analyzed. Images acquired from two independent experiments. *p*-values determined by Mann-Whitney; ns = non-significant, * = *p* < 0.05, **p < 0.01, ***p < 0.001.

Phagoctyosis requires force generation mediated through F-actin, vinculin and Pyk2 (Duong and Rodan, 2000; Jaumouillé et al., 2019). Vinculin forms a “molecular clutch” during phagocytosis, enabling integrin-mediated retrograde flow that supports internalization of particles (Jaumouillé et al., 2019). To evaluate possible mechanisms by which S5A-LPL might disrupt phagocytosis, we evaluated co-localization of vinculin and Pyk2 with internalized pneumococcus. Aggregates of Spn are used to define sites of phagocytosis, or phagosomes. Notably, the podosome marker Tks5 did not co-localize with ingested Spn in WT AMs (Supplementary Figure S6). While individual aggregates of F-actin were similar in AMs expressing either WT or S5A-LPL, the volume of vinculin aggregates was profoundly reduced (Figure 10A). Aggregates of co-localized F-actin + vinculin, F-actin + Spn, vinculin + Spn, and F-actin + vinculin + Spn were also significantly reduced in AMs expressing S5A-LPL (Figure 10A). Reduced recruitment or inclusion of vinculin at sites of phagocytosis could partially explain impaired phagocytosis when S5A-LPL is expressed.

In contrast to AMs expressing S5A-LPL, LPL-deficient AMs exhibited volumes of F-actin and F-actin + Spn aggregates comparable to those of B6 (Figure 10B). Volumes of aggregates of vinculin and of co-localized F-actin + vinculin and vinculin + Spn were increased by LPL-deficiency (Figure 10B). Thus, the incorporation of vinculin into sites of phagocytosis appears to be negatively regulated by WT-LPL.

**FIGURE 11:**
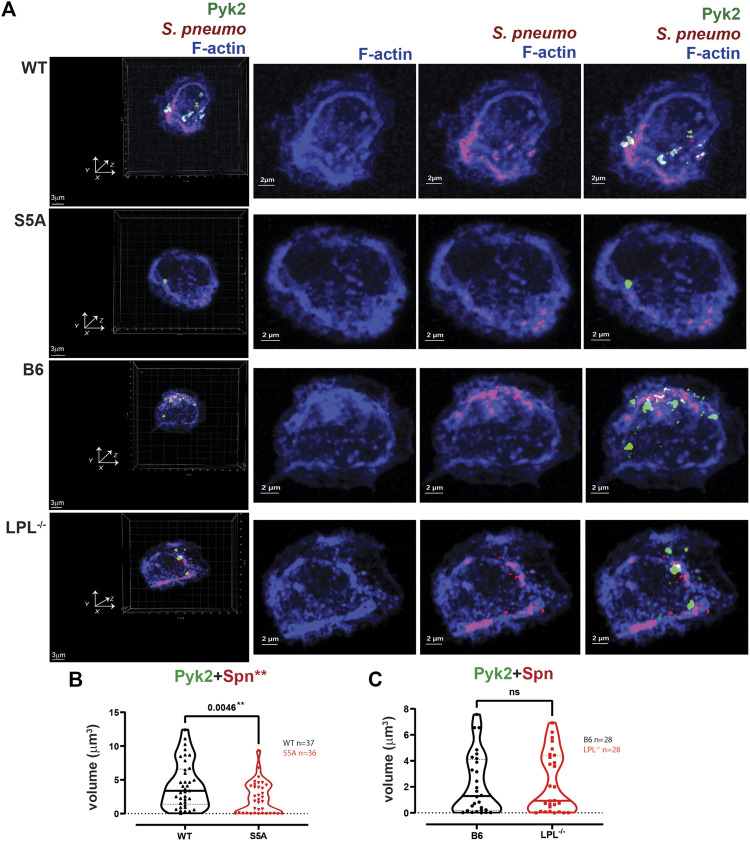
Quantification of volumes of protein aggregates reveals loss of Pyk2 from phagosomes in AMs expressing S5A-LPL. **(A)** Co-localization of Pyk2 (green), *S. pneumoniae* (red) and F-actin (blue) in AMs incubated with Spn-RFP for 60 min from matched WT and S5A or matched B6 and LPL^−/−^ mice. Images in left panels show 3D reconstruction of Z-stack confocal micrographs. Other panels are 2D XY projections showing F-actin only, Spn overlaid with F-actin, or overlay of Pyk2+Spn + F-actin. Phagosomes incorporate F-actin, Spn and Pyk2 (white; representative phagosomes indicated by white arrows). Grids show 2 μm increments and are optimal for size assessment; scale bars in subsequent panels are derived from grids. **(B, C)**. Total volumes of aggregates of Pyk2+Spn from WT and S5A samples **(B)** or B6 and LPL^−/−^ samples **(C)**. Each symbol represents the volume calculated from one fixed cell; n = number of cells analyzed. Images acquired from two independent experiments. *p*-values determined by Mann-Whitney; ns = non-significant; **p < 0.01.

Pyk2 supports CR3-mediated phagocytosis (Paone et al., 2016), possibly by recruiting vinculin. We therefore also analyzed the localization of Pyk2 during pneumococcal phagocytosis (Figure 11). The volume of aggregates of Pyk2-Spn was significantly reduced in AMs expressing S5A-LPL (Figures 11A, B), consistent with reduced phagocytosis in AMs from S5A mice. Volumes of Pyk2-Spn aggregates were equivalent in AMs from LPL^−/−^ mice, compared to B6 controls (Figures 11A, C).

**FIGURE 12:**
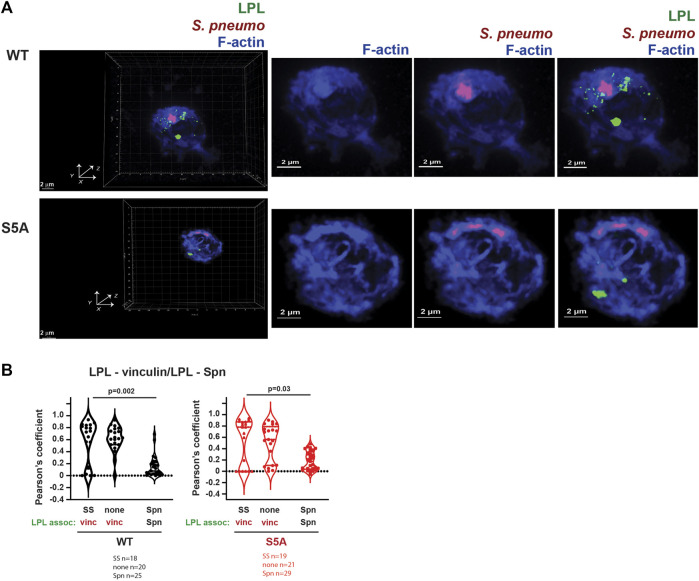
Neither WT-LPL nor S5A-LPL significantly co-localized with phagosomes in AMs. **(A)** Visualization of LPL (green), *S. pneumoniae* (red) and F-actin (blue) in AMs from matched WT and S5A mice after incubating with Spn-RFP for 60 m. Images in left panels show 3D reconstruction of Z-stack confocal micrographs. Other panels are 2D XY projections showing F-actin only, F-actin + Spn (purple), and LPL + F-actin + Spn. Grids show 2 μm increments and are optimal for size assessment; scale bars in subsequent panels are derived from grids. **(B)** Pearson’s coefficient was calculated for LPL and vinculin (Figures 4, 5, 8) or for LPL and Spn (Figure 12A). Each symbol represents the coefficient for one cell. “SS” = serum stimulation, “none” = incubation for 60 min without additional stimulation; “Spn” = incubation with Spn-RFP. *p*-values determined by Mann-Whitney.

Finally, we evaluated if LPL co-localized with pneumococcal phagosomes by imaging AMs from WT and S5A mice after incubation with Spn-RFP. Images did not show significant co-localization of WT-LPL with Spn, suggesting that LPL is not incorporated into phagosomes (Figure 12A). To quantify the degree of co-localization of LPL with Spn, we measured Pearson’s coefficient. Pearson’s coefficient measures the degree to which two signals co-localize within a given image and is independent of signal intensity. A coefficient of 1 indicates perfect overlay (perfect co-localization), a coefficient of 0 indicates random distribution of the two signals, and a coefficient of −1 indicates perfect separation (no co-localization) of the two signals. Because the localization of vinculin to podosomes does not require LPL, and because both vinculin and LPL are recruited to podosomes, we used Pearson’s coefficient in podosome-forming AMs as a “positive control” for LPL recruitment to a vinculin-inclusive, F-actin structure.

In AMs expressing WT LPL or S5A-LPL, Pearson’s coefficients for colocalization of LPL with vinculin are largely positive under conditions in which podosomes are forming (Figure 12B). In contrast, the Pearson’s coefficients for WT LPL and Spn cluster around zero, and are significantly reduced compared to the Pearson’s coefficients of LPL + vinculin after serum stimulation. Pearson’s coefficients near zero suggest that LPL and Spn are randomly distributed in cells, relative to each other, consistent with a model in which LPL is not recruited to sites of phagocytosis. Quantification of Pearson’s coefficients for S5A-LPL and Spn is also significantly reduced compared to the coefficient of S5A-LPL co-localization with vinculin (Figure 12B).

Our systematic and rigorous analysis of podosome formation and phagocytosis in AMs derived from WT, S5A and LPL^−/−^ mice has revealed differential requirements for LPL expression and LPL S5 phosphorylation in the formation and recruitment of essential mediators vinculin and Pyk2. (Table 1). Use of the primary AM lineage enables extrapolation to physiologically relevant applications, such as pneumococcal lung infection. Further study on how different macrophage lineages rapidly remodel F-actin for adhesion, motility and host defense can illuminate multiple pathophysiological processes.

**TABLE 1:** Expression of S5A-LPL exerts different effects on nascent podosome initiation compared to LPL deficiency.

Stimulation	Variable assessed	WT/B6	S5A-LPL	LPL-deficient
Serum-stimulated	Number	ctrl	─	↓
	F-actin	+++	+++	+
	Vinculin	+++	+++	**+++**
	Pyk2	+++	+++	+
	LPL	+++	+++	*absent*
60 min culture	Number	ctrl	↓	↓
	F-actin	+++	+	+
	Vinculin	+++	+	+
	Pyk2	+++	+	+
	LPL	+++	+++	*absent*
*S. pneumoniae*	Volume ingested	ctrl	↓	─
	F-actin	+++	+	+++
	Vinculin	+++	+	+++++
	Pyk2	+++	+	+++
	LPL	not recruited	not recruited	*absent*

This table summarizes the results obtained from the imaging experiments presented here. Under the 3 conditions analyzed, AMs from matched WT or B6 mice are used as the control (“ctrl”) or baseline (“+++”) for aggregation of the indicated proteins to sites of adhesion or ingested S. pneumoniae. LPL deficiency resulted in diminished nascent podosome numbers and reduced F-actin aggregation after serum stimulation and after 60 min incubation, but did not impair phagocytosis. In contrast, expression of S5A-LPL disrupted phagocytosis, but not nascent podosome initiation after serum stimulation. Relative reductions (↓ or +) or increases (+++++) in co-aggregation of the analyzed proteins are shown; “─” indicates no change.

## Discussion

Rapid rearrangement of the actin cytoskeleton drives key immune processes, including adhesion, motility and phagocytosis. Actin filaments are generated through the rapid polymerization of the G-actin monomers, and remodeled through depolymerization, severing, and capping. Scores of actin-binding proteins regulate F-actin remodeling through monomer sequestration, nucleation, nucleotide exchange, branching, cross-linking, capping, and severing. Furthermore, actin-based structures enable sensation and generation of mechanical forces. For instance, podosomes are large, multimolecular complexes formed at the site of integrin-mediated adhesion, and are mechanosensing and mechanotransducing. Integrin adhesion can also mediate phagocytosis, an actin-dependent process that requires generation of mechanical forces to engulf and enclose foreign particles.

While essential to immunity, the molecular mechanisms regulating actin rearrangement and force generation during adhesion and phagocytosis have not been fully elucidated, nor have all the requisite actin-binding proteins been identified. In prior, separate studies, we had observed that peritoneal macrophages lacking LPL exhibited unstable podosomes, but that LPL appeared dispensable for phagocytosis by neutrophils and splenic macrophages (Deady et al., 2014; Todd et al., 2016; Zhou et al., 2016). Intriguingly, expression of endogenous LPL that could not be phosphorylated on residue serine 5 inhibited phagocytosis, but did not appear to destabilize podosomes in peritoneal macrophages (Anaya et al., 2021). However, these studies had been carried out in multiple macrophage lineages, and the recruitment of additional actin-binding or signaling proteins to podosomes or phagosomes in the presence or absence of WT or S5A-LPL had not been rigorously evaluated.

To systematically investigate the participation of WT-LPL and S5A-LPL in podosome formation and in phagocytosis, we analyzed high-resolution confocal images of fixed primary AMs from WT, S5A, B6 or LPL^−/−^ mice under three conditions: 30 min following exposure to serum after 30 min serum starvation (“serum stimulated,”), 60 min of incubation on coverslips after isolation but without additional stimulation (“60 min incubation”), and 60 min after incubation with fluorescent *S. pneumoniae* serotype D39 (“Spn”). Because prior work had suggested that the kinase Pyk2 was mislocalized in the absence of LPL, but that the adaptor protein vinculin was not, and because both Pyk2 and vinculin support podosome formation and phagocytosis, we quantified the co-localization of F-actin with Pyk2 and vinculin at podosomes and sites of Spn internalization (Table 1).

Our results fully support our prior findings that LPL maintains podosome stability, but is dispensable for phagocytosis: podosome size and numbers were greatly reduced in AMs derived from LPL^−/−^ mice, but ingestion of Spn was unimpeded. In contrast, AMs derived from mice expressing S5A- LPL showed reduced Spn ingestion, indicating disruption of phagocytosis, but nascent podosome initiation after serum stimulation appeared largely intact. We extended our prior mechanistic analysis of serum-stimulated podosome formation in the presence or absence of LPL by first recapitulating our prior findings that LPL deficiency disrupts Pyk2, but not vinculin, co-localization with F-actin (Figures 2–5). We also observed reduced overall aggregation of F-actin and of vinculin, consistent with reduced stability of nascent podosomes in the absence of LPL. We note that in prior work using live-cell imaging, we observed a similar number of podosomes initiate formation in peritoneal macrophages from LPL^−/−^ mice, but reduced stability resulted in overall reduced numbers and size of podosomes 30 min after serum stimulation (Zhou et al., 2016). Reduced aggregates of F-actin and of vinculin in LPL-deficient AMs after serum stimulation is consistent with our prior work in peritoneal macrophages (Zhou et al., 2016). Expression of S5A-LPL did not significantly reduce recruitment of Pyk2 to podosomes after serum stimulation (Figures 2, 5), correlating with similar numbers of nascent podosomes after serum-stimulation, compared to WT.

Nascent podosomes form in AMs expressing WT-LPL when incubated on glass coverslips immediately after isolation, presumably due to adhesion to a stiff substrate and independently of additional extracellular stimulation. Under these conditions, expression of S5A-LPL also reduced nascent podosome formation (Figures 6–8). Interestingly, Tks5 co-localization to aggregates of F-actin/vinculin/Pyk2 or F-actin/vinculin/LPL did not appear as robust in AMs expressing S5A-LPL as in WT AMs (Supplementary Figures S4, S5), consistent with reduced stability or possibly maturation of true podosomes in AMs expressing S5A-LPL. It is not yet clear if the absence of podosomes in S5A AMs is due to a failure to initiate podosome formation in response to the presumed adhesive stimulus, or if podosomes are unstable and disassemble more rapidly during the 60 min incubation. These possibilities require future live-cell imaging and higher resolution imaging for complete elucidation.

Further investigation of how LPL and S5 phosphorylation support macrophage podosome formation is warranted, in part, because of the similarity of podosomes to cancer cell invadopodia. Like podosomes, invadopodia are F-actin-based, mechanotransducing cellular protusions that mediate adhesion and migration. LPL has been shown to be a component of invadopodia in some cancer cells (Van Audenhove et al., 2016), and S5 phosphorylation of LPL promotes incorporation of LPL into invadopodia (Machado et al., 2021). LPL has been proposed to contribute to matrix degradation and invadopodia length and stability (Van Audenhove et al., 2016); it would be fruitful to determine if LPL and its phosphorylation status contributes similarly to macrophage podosomes.

Analysis of recruitment of vinculin and Pyk2 to phagosomes in AMs expressing S5A-LPL or lacking LPL revealed significant differences compared to phagosomes formed with WT LPL. Phagosomes were defined as aggregates of internalized Spn. In WT AMs, both vinculin and Pyk2 co-localized with internalized Spn, as expected. In the absence of LPL, there was a significant increase in the co-localization of vinculin with F-actin and Spn (Figure 10B), while Pyk2+Spn volumes were equivalent to WT. In contrast, the volumes of vinculin + Spn aggregates were profoundly diminished in the presence of S5A-LPL. Vinculin + Spn aggregates were diminished more than aggregates of F-actin + Spn, suggesting that less vinculin was co-localizing with internalized Spn (Figure 10A). Pyk2 co-localization at sites of internalized Spn may also have been reduced by expression of S5A-LPL (Figure 11). Our results highlight that ablation of the S5 phosphorylation site on LPL is not functionally equivalent to LPL deficiency. Furthermore, expression of S5A-LPL can uncover unexpected consequences of altered LPL function during immune processes.

Our study had several technical limitations. First, we lack an antibody specific for S5-phosphorylated LPL that can be used for immunofluorescent imaging. While the currently available antibodies specifically illuminated S5-phosphorylated LPL in immunoblots, they were unreliable in our system for fluorescent imaging. LPL can also be phosphorylated on S7, and thus an anti-phosphoserine antibody cannot be used for imaging specifically S5-phosphorylated LPL. Our study was also limited by the inability to immunoprecipitate LPL using currently available antibodies. While we can immunoprecipitate Pyk2, we have not been able to directly detect associated LPL by immunoblot. It is possible that Pyk2 and LPL associate in the F-actin-associated, detergent-insoluble fraction, rendering co-immunoprecipitation from the cytosolic, detergent-soluble fraction impossible. Our available imaging modality (Airyscan) is of insufficient resolution to define the nascent podosome structures as clearly as STORM or PALM could (Hu et al., 2022), and the resolution of our Airyscan could preclude clear delineation of “ring-and-core” appearances of podosomes. In our system, most nascent podosomes appeared as punctae, not ring-and-core, which has been previously been seen when primary macrophages are incubated *in vitro* for relatively brief durations (≤4 h) (Hu et al., 2022). We did not incubate AMs for longer *in vitro* for podosome analysis, as we wished to directly compare changes in F-actin structures following exposure to Spn infection, and prolonged exposure to Spn induces AM death. Future work in which additional macrophage lineages are incubated *in vitro* for longer durations to permit podosome maturation may illuminate more significant podosome disruption in cells expressing S5A-LPL. Finally, we are unable to exclude the possibility that AMs that are actively phagocytosing Spn may also form podosomes (or even “phagocytic podosomes”) (Hu et al., 2022), which would confound some of the volume measurements. However, we did not observe many (if any) structures that resembled podosomes in AMs challenged with Spn: further work to specifically address if AMs can form phagosomes and podosomes simultaneously is warranted.

In summary, we have systematically analyzed podosome formation and phagocytosis in AMs derived from WT, S5A, or LPL^−/−^ mice. LPL is required for podosomes, but dispensable for phagocytosis. Expression of S5A-LPL can rescue podosomes under some, but not all conditions, suggesting a role for S5-phosporylation in podosome initiation, stability, or turnover that should be elucidated in future work. Expression of S5A-LPL also inhibits phagocytosis, and impairs inclusion of both vinculin and Pyk2 to sites of phagocytosis. Our work highlights how specific recruitment of select actin-binding proteins controls the rapid cytoskeletal remodeling required for effective immunity.

## Data Availability

The original contributions presented in the study are included in the article/Supplementary Material, further inquiries can be directed to the corresponding author.
