# Supplementary material for: Expression of non-phosphorylatable S5A-L-plastin exerts phenotypes distinct from L-plastin deficiency during podosome formation and phagocytosis
Source: Front Cell Dev Biol. 2023 Apr 17;11:1020091. doi: 10.3389/fcell.2023.1020091 (PMC10150066; doi:10.3389/fcell.2023.1020091)
Supplement: Supplementary file 2 [file DataSheet1.pdf]

Supplemental Figures

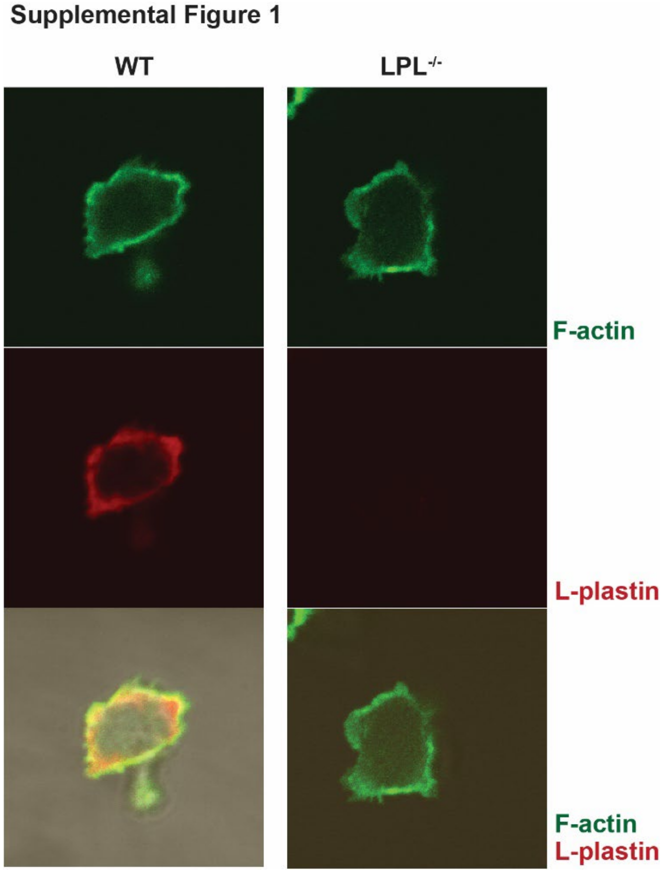

Supp. Fig. 1. Validation of anti-LPL mAb for immunofluorescence. T cells isolated from WT or LPL$^{-/-}$ mice were incubated on coverslips coated with ICAM-1, then stimulated with CCL19 and fixed. After permeabilization, LPL was visualized by staining with anti-LPL mAb, and actin was visualized by staining with phalloidin-488. T cells from LPL$^{-/-}$ mice show the specificity of anti-LPL mAb staining. From Morley et al., 2010, J Immunol 184(7):3628-38.

Supplemental Figure 2

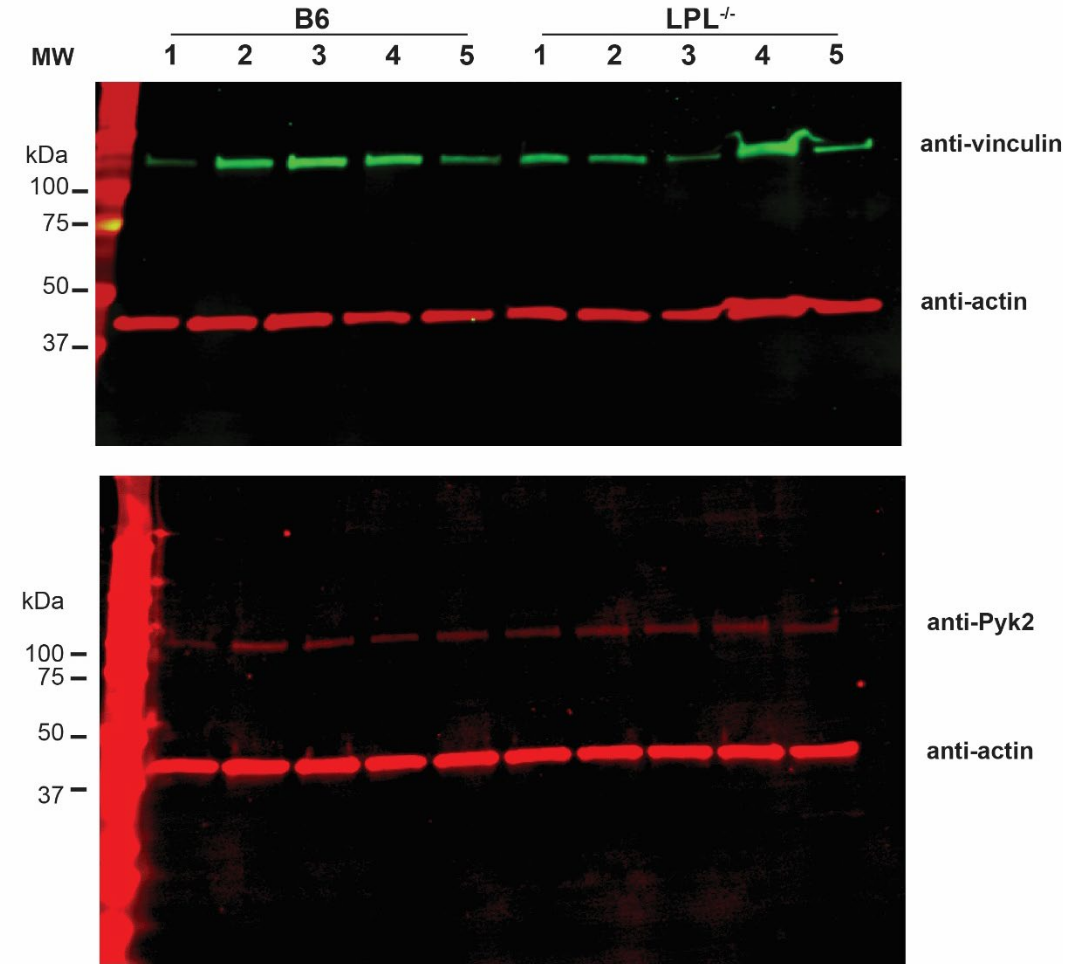

Supp. Fig. 2. Specificity of mAb used in this manuscript. Imaging immunoblots with the LiCOR Odyssey system uses infrared dyes to label secondary antibodies, permitting visualization of primary antibodies from different species (in this example, anti-vinculin and anti-actin) on two different channels, demonstrating that each antibody illuminates only one specific band of the appropriate molecular weights. Once the anti-actin mAb was validated, it could then be imaged simultaneously with a primary anti-Pyk2 antibody, which also illuminates only one protein band of the appropriate molecular weight. Simultaneous imaging of the protein of interest with the loading control ensures optimal normalization for loading. Please note that these images are the same immunoblot images as those in Supp. Fig. 3.

Supplemental Figure 3

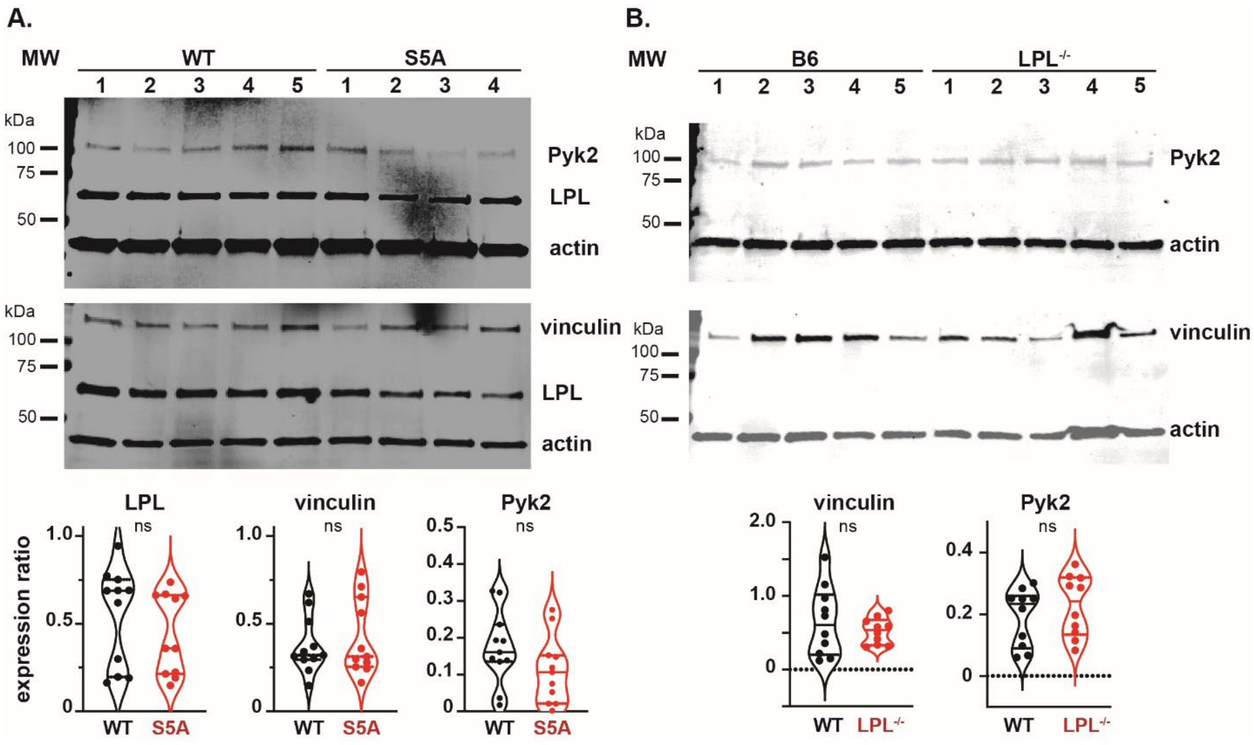

Supp. Fig. 3. Equivalent protein expression of vinculin and Pyk2 in AMs derived from WT vs. S5A and B6 vs. LPL$^{-/-}$ mice. Immunoblots of post-nuclear lysates of AMs derived from (A) matched WT and S5A or (B) matched B6 and LPL$^{-/-}$ mice and probed for Pyk2, vinculin, actin, and/or LPL. Densitometric quantification of protein expression, normalized to actin, shown below panels. Each symbol represents data from one mouse; data combined from two independent experiments containing 5-6 mice of each genotype. Please note that these images are the same immunoblot images as those in Supp. Fig. 2. P-values measured using Mann-Whitney, ns = not significant.

Supplemental Figure 4

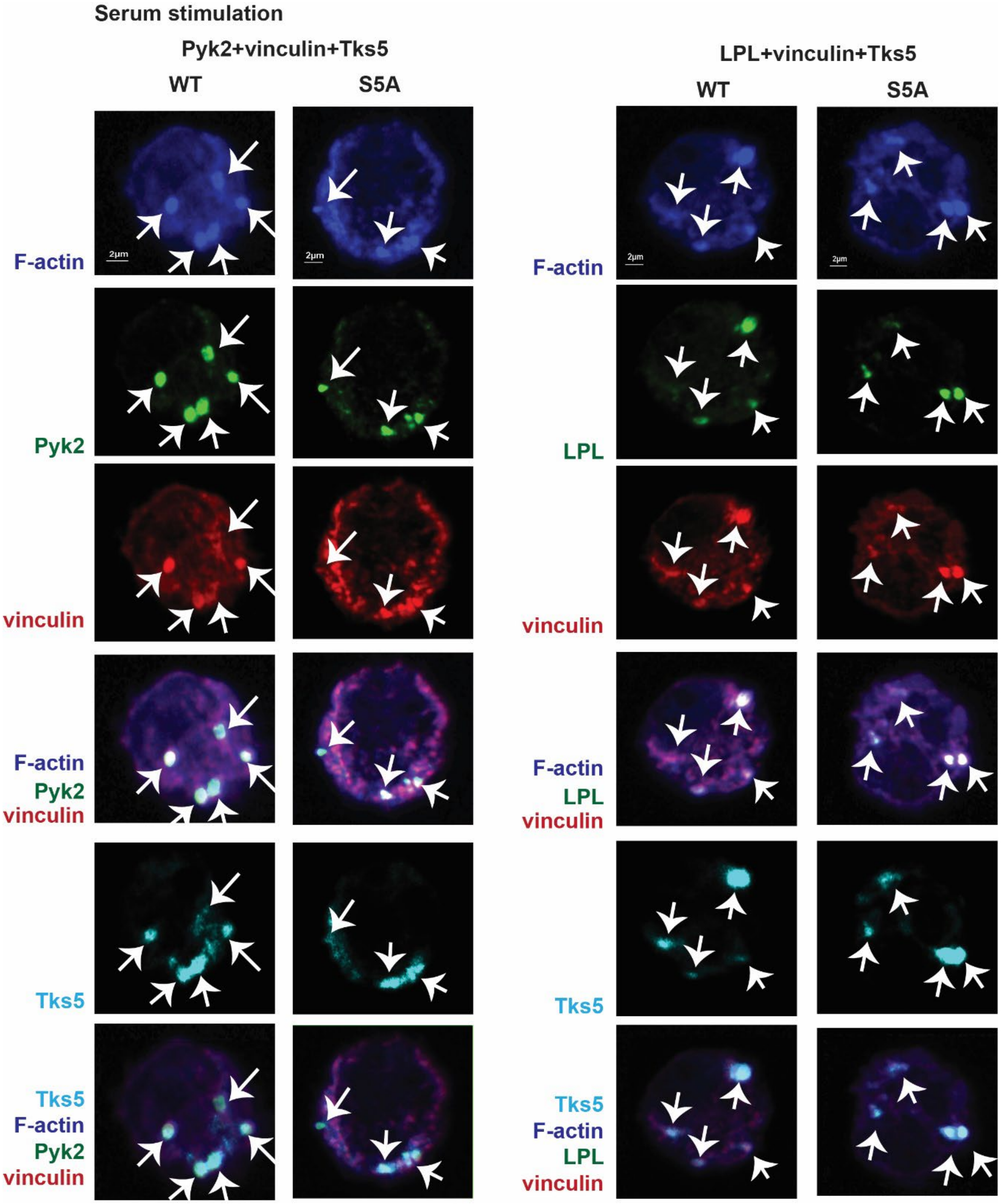

Supp. Fig. 4. The scaffold protein Tks5 co-localizes with nascent podosomes defined as co-aggregates of F-actin/Pyk2/vinculin and of F-actin/LPL/vinculin. Tks5 is a specific marker for podosomes and invadopodia. AMs were prepared as in Fig. 1, 2, 4 and 5 with incubation in

medium containing 1% FBS for 30 min followed by incubation in medium containing 10% FBS. After fixation, the indicated proteins were stained and visualized by Airyscan confocal imaging. Shown are 2D X-Y projections of full-thickness z-stacks. Arrows indicate examples of co-aggregates identified as “nascent podosomes.” To best exemplify overlay, the Tks5-only image is directly overlaid the combined F-actin+Pyk2+vinculin or F-actin+LPL+vinculin images with 50% transparency (PhotoShop). Images representative of 3 AMs each. Scale bars indicate 2 μm.

Supplemental Figure 5

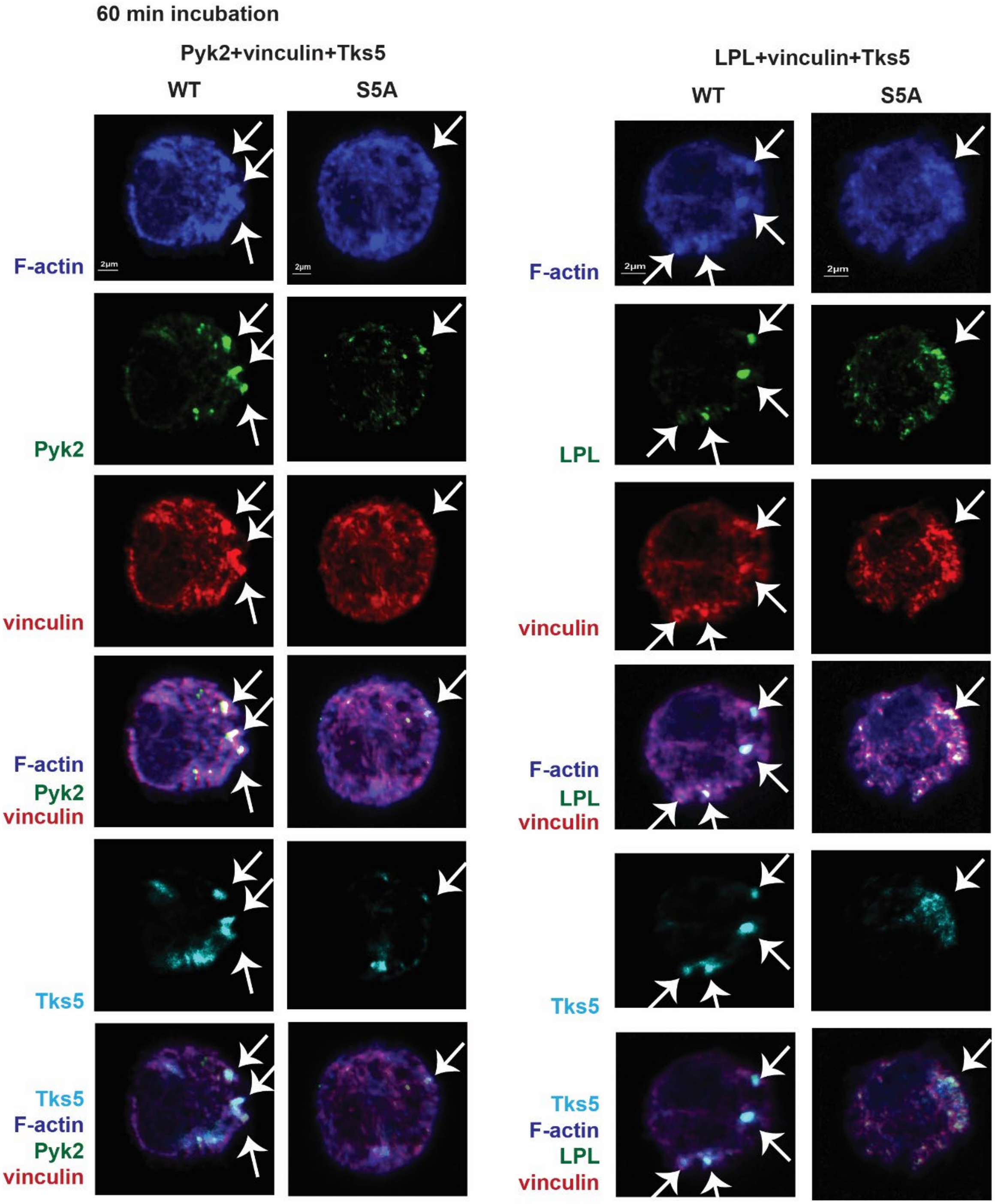

Supp. Fig. 5. Tks5 co-localizes with nascent podosomes, defined as co-aggregates of F-actin/Pyk2/vinculin and of F-actin/LPL/vinculin. AMs were incubated in medium containing 10% FBS for 60 min (no specific stimulation), as in Fig. 6. After fixation, the indicated proteins

were stained and visualized by Airyscan confocal imaging. Shown are 2D X-Y projections of full-thickness z-stacks. Arrows indicate examples of co-aggregates identified as "nascent podosomes." To best exemplify overlay, the Tks5-only image is directly overlaid the combined F-actin+Pyk2+vinculin or F-actin+LPL+vinculin images with 50% transparency (PhotoShop). Images representative of 3 AMs each. Scale bars indicate 2 μm.

Supplemental Figure 6

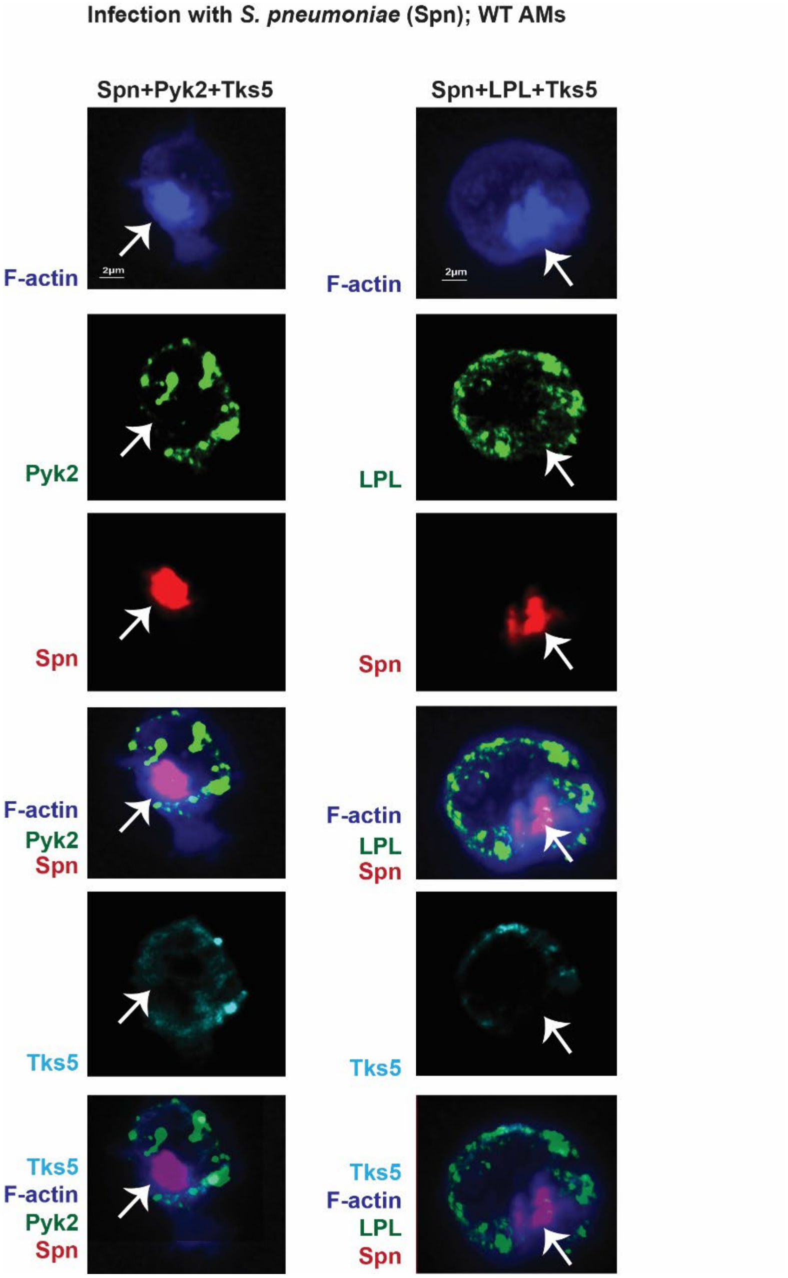

Supp. Fig. 6. Tks5 does not co-localize with internalized bacteria. AMs were incubated with Spn-RFP for 60 min, as in Fig. 9. After fixation, the indicated proteins were stained and visualized by Airyscan confocal imaging. Shown are 2D X-Y projections of full-thickness z-stacks. Arrows indicate areas of ingested Spn, identified as "phagosomes." To best exemplify overlay, the Tks5-only image is directly overlaid the combined F-actin+Pyk2+vinculin or F-

actin+LPL+vinculin images with 50% transparency (PhotoShop). Images representative of 3 AMs each. Scale bars indicate 2 μm.

Supplemental Videos

Supp. Videos 1-4. Representative z-stacks of AMs captured by Airyscan confocal microscopy and reconstructed as 3D projections using Imaris software. All videos depict AMs isolated from the mice indicated below that underwent serum stimulation (cells presented in Fig. 2). F-actin (blue), vinculin (red) and Pyk2 (green), along with nuclear staining (DAPI; blue), were visualized. Overlaid grid provided for scale (intervals listed below). Axis labels and scale bars provided in Fig. 2.

Supp. Video 1: WT; large ticks at 2 μm intervals
Supp. Video 2: S5A; large ticks at 2 μm intervals
Supp. Video 3: B6 large ticks at 3 μm intervals
Supp. Video 4: LPL$^{-/-}$; large ticks at 3 μm intervals
